# The first WHO reference panel for Infliximab anti-drug antibodies: a step towards harmonizing therapeutic drug monitoring

**DOI:** 10.3389/fimmu.2025.1550655

**Published:** 2025-03-20

**Authors:** Meenu Wadhwa, Isabelle Cludts, Eleanor Atkinson, Peter Rigsby

**Affiliations:** ^1^ Biotherapeutics and Advanced Therapies Group, R&D Division, Science and Research, Medicines and Healthcare Products Regulatory Agency (MHRA), South Mimms, United Kingdom; ^2^ Analytical and Biological Sciences Group, R&D Division, Science and Research, Medicines and Healthcare products Regulatory Agency (MHRA), South Mimms, United Kingdom

**Keywords:** binding, calibration, clinical monitoring, reference standard, assay performance, anti-drug antibodies, patient, infliximab

## Abstract

Immunogenicity testing for anti-drug antibodies (ADA) is mandatory for regulatory approval of a biotherapeutic and can, in some instances, continue post-licensure. Typical examples are TNF inhibitors where biotherapeutic and ADA levels are relevant in clinical decision-making for optimal patient therapy. However, challenges with non-comparability of results due to plethora of bioanalytical techniques and the lack of standardization has hindered ADA monitoring in clinical practice. Two human anti-infliximab monoclonal antibodies (A, B) with defined characteristics were therefore lyophilized and assessed for suitability as a reference panel for ADA assays in an international study. Binding assays included the simple ELISA and common electrochemiluminescence (ECL) to the rare antigen binding test and lateral flow assays. For neutralisation, competitive ligand binding and reporter-gene assays were employed. Sample testing (e.g., antibodies, sera) showed differential reactivity depending on the assay and sample. Estimates for ADA levels using in-house standards varied substantially among assays/laboratories. In contrast, using antibody A for quantitating ADA levels reduced the interlaboratory variability and provided largely consistent estimates. The degree of harmonization was dependent on the assay, sample and the laboratory. Importantly, antibody A allowed ADA detection when missed using in-house standards. Recognition of sample B varied, possibly due to its fast dissociation. Overall, the panel comprising A (coded 19/234) and B (coded 19/232) was suitable and established by the WHO Expert Committee on Biological Standardization in October 2022 as the WHO international reference panel for infliximab ADA assays. Sample A (coded 19/234) with an arbitrarily assigned unitage of 50,000IU/ampoule for binding activity and 50,000 IU/ampoule for neutralising activity is intended as a ‘common standard’ for assay characterization and where possible for calibration of anti-infliximab preparations to facilitate comparison and harmonization of results across infliximab ADA assays. Sample B (19/232) with its unique characteristics and variable detection but no assigned unitage is intended for assessing the suitability of the assay for detecting ADAs with fast dissociation. It is anticipated that this panel would help towards selecting and characterizing suitable assays, benchmarking of in-house standards where feasible and in harmonizing ADA assays used in clinical practice for better patient outcome globally.

## 1 Introduction

The approval of the first anti-tumor necrosis factor (TNF) monoclonal antibody, infliximab (IFX, Remicade^®^, Janssen; US in 1998, EU in 1999) radically changed treatment strategies for rheumatoid arthritis and for other chronic immune and inflammatory disorders. Despite its high costs and limited patient access, Remicade^®^ achieved clinical success and block-buster global sales. Loss of patent exclusivity, however led to the European approval of the first biosimilar IFX (Remsima^®^/Inflectra^®^, Celltrion Inc/Hospira in 2013) followed by other biosimilars worldwide which is contributing to a decrease in drug costs, in widening patient access and is significantly transforming disease management across different indications.

Despite the demonstrated clinical benefits of TNF inhibitors, a certain proportion of patients are non-responsive to treatment (primary failure) or fail to maintain an adequate response after initial improvement (secondary failure) and/or develop adverse events which limits treatment with infliximab (1–8). In Crohn’s disease, 10-30% of patients are non-responsive and up to 60% of patients lose response to anti-TNF therapy over time, potentially due to development of anti-drug antibodies (ADA) (9). Such ADAs primarily target the TNF binding region (10), prevent the drug from binding to TNF and neutralize its bioactivity reducing the drug’s clinical efficacy. Drug-ADA immune complexes can also arise and cause accelerated drug clearance (8, 11). Given the potential for ineffective treatment and clinical sequelae (11), immunogenicity is of concern in the clinic and, therefore, treatment with infliximab may require implementation of therapeutic drug monitoring (TDM) for effective patient management.

Published evidence indicates variation in the reported frequencies of ADA detection as well as in ADA titers between studies (12). In addition to the product, differences in patient characteristics (including gene susceptibility, e.g., carriage of human leukocyte antigen [HLA]-DQA1*05 risk variant for infliximab (13, 14), the disease state and treatment-related factors such as mode of administration, co-medication with immunomodulators, duration of follow-up and dosing regimen contribute to the immunogenicity (15). Furthermore, sampling times and assay format employed for ADA assessment also influence the results (12, 16). Importantly, the clinical impact depends on the balance achieved between the relative amount of drug and ADA i.e. the ratio between the amount of drug neutralized by ADA and the amount of free drug. In some cases, drug levels are sufficiently high and, despite ADA formation can contribute to clinical remission while in others, ADAs diminish drug levels substantially and lead to treatment failure. As a result, therapeutic drug monitoring (TDM) which includes not only monitoring of drug levels but also ADA and associated outcomes, is an important consideration in the clinic (7, 13, 15, 17–19).

TDM has the potential to improve clinical decision-making for patients, by influencing dose selection, frequency of administration, and possibly even an earlier switch to another therapeutic for optimal treatment. Some clinical laboratories have implemented TDM in health care by employing commercial kits or methods developed in-house but others remain cautious due to conflicting or non-comparable results often due to use of assays with different characteristics (20) and the lack of standardization (7, 19, 21). In recognition of this need, the World Health Organization (WHO) which has a core role in developing norms and standards for biological medicines has established WHO International standards (IS) for monitoring levels of some biotherapeutics (22). For example, WHO IS for Infliximab, Adalimumab and Golimumab (23–25) are available through the UK’s Medicines and Healthcare products Regulatory Agency, MHRA (which has taken over the responsibility for standardization from National Institute for Biological Standards and Control, NIBSC, a WHO collaborating center which produces and distributes 95% of international standards, IS for biological medicines and vaccines). However, the recognition by clinicians of the need to standardize ADA testing across different analytical assay platforms/laboratories has remained largely unfulfilled (7, 26–28).

In the development and validation of ADA assays associated with immunogenicity testing for biotherapeutic approval, positive controls (PC) serve a critical role (e.g., for determining assay sensitivity, selectivity, specificity, drug interference). PCs are also used for quality control (QC) purposes and allow for assay performance monitoring during life-cycle management. Long-term provision of suitable PCs with properties which support the capabilities of different assays e.g., binding, neutralizing activity is essential. The provision of PCs in assuring the analytical performance of the different ADA tests for clinical monitoring and in facilitating the immunogenicity assessment of emerging biosimilar medicines (29) aligns with the World Health Assembly Resolution on access to safe and effective medicines (30). To achieve this goal, we initiated a collaboration with stakeholders – clinicians, diagnostic laboratories and regulators to develop positive controls/reference standards for ADA assays.

This article describes the strategy employed for the development of the 1^st^ WHO international reference panel for infliximab anti-drug antibodies, following WHO endorsement (31) based on global need and priority. Results from a large international collaborative study with participation from various stakeholders is also presented. The data generated in this study illustrates the need and the suitability of the lyophilized antibody preparations to serve as the 1^st^ WHO international reference panel for infliximab anti-drug antibodies across different assays. This article highlights the applicability and role of the two components of the infliximab anti-drug antibody panel in assays for clinical monitoring of infliximab ADAs.

## 2 Materials and methods

### 2.1 Materials, processing and characterization

Two human mAbs against infliximab, INA29 and INA79, expressed in CHO cells were kindly donated by the ABIRISK consortium, funded by the Innovative Medicines Initiative, EU (2012-2017). The characteristics of these mAbs isolated and cloned from memory B cells separated from cryopreserved peripheral blood mononuclear cells (PBMCs) of an infliximab treated patient are provided in **Table 1**. A brief outline of the production method and antibody characterization methods (32, 33) are described in **Supplementary Data Sheet 1**.

**Table 1:** Characteristics of the antibodies sourced for the infliximab ADA reference panel.

Antibody	Origin	Clone	Isotype	Light chain	Binding		Neutralisation	
					Affinity EC50 (ng/ml)	KD (M) SPR	Status	Activity EC90 (ng/ml)
INA29	human PBMC	VA2-17-478-1	IgG1	κ	9	3 E-10	+ve	66
INA79	human PBMC	VA2-17-479-1	IgG4PE^1^	κ	12	1.7 E-10	+ve	489

INA29 and INA79 sourced from ABIRISK. ^1^PE represents mutations commonly introduced in the IgG4 antibody (P and E @ positions 228 and 235 for increased stability and reduced binding to Fcγ receptors for depletion of effector functions respectively). Affinity of the antibodies is expressed as EC50: concentration inducing a response halfway between baseline and maximum and KD: dissociation constant (k_off_/k_on_) as determined by ELISA and SPR (ProteOn, Biorad, US) respectively. Neutralization activity is expressed as EC90: concentration giving 90% of E_max_ and determined by competitive ligand binding assay.

The materials were formulated and freeze-dried using different formulations (33) and tested in binding and neutralization assays in-house along with stability testing at 3 months to select a suitable formulation for lyophilization. Details of different formulations tested are described (33). Since the formulation containing 10mM L-Glutamic acid, 4% Mannitol, 2% Sucrose, 0.01% Tween 20 pH 5.2 did not induce aggregate formation as detected by SE-HPLC and showed similar binding profile and neutralisation activity as the bulk antibodies, it was chosen for the manufacturing of the final lyophilized preparations (data not shown).

The final lyophilization of both antibodies was performed at MHRA using standardized procedures as specified in the WHO ECBS recommendations for IS (34). Antibody solutions with excipients were prepared using nonpyrogenic water for irrigation (Baxter, Switzerland) and filtered using sterile nonpyrogenic filters (0.22μM Stericup filter system, Millipore, USA). For both mAbs, a solution of ~ 50μg/ml given as ‘predicted μg’ in **Table 2**, calculated from the dilution of the bulk material of known protein mass content, was distributed in 1 ml aliquots into 5 ml ampoules using an automated filling line (Bausch and Stroebel, Ilshofen, Germany) and freeze-dried. Optimized and controlled conditions were used for lyophilization and the glass ampoules sealed under dry nitrogen by heat fusion with storage at -20°C in the dark until shipment.

**Table 2:** Characteristics of lyophilized infliximab ADA preparations.

Ampoule code	Study code	Protein (predicted μg)	Fill Weight		Residual moisture		Headspace Oxygen	
			Mean g (n)	CV%	Mean % (n)	CV%	Mean % (n)	CV%
19/234	A	50	1.0082 (65)	0.20	1.23 (12)	28.7	0.31 (12)	32.1
19/232	B	50	1.0089 (96)	0.33	1.27 (12)	32.5	0.41 (12)	35.6

Both preparations (CHO cell-expressed) were formulated using 10mM L-Glutamic acid, 4% Mannitol, 2% Sucrose, 0.01% Tween20, pH 5.2. Both are stored at -20°C at MHRA which is the custodian and distributor laboratory.

CV, Coefficient of Variation; n, number of determinations. Residual moisture of each preparation was measured by the coulometric Karl-Fischer method (Mitsubishi CA100). Headspace oxygen content was determined by frequency modulated spectroscopy (Lighthouse FMS-760). Protein content of lyophilized preparations was determined using a spectrophotometer and confirmed to be 50 μg.

**Table 2** provides the characteristics of the lyophilized mAbs and study codes. For both mAbs, the WHO specifications for standards were met. Ampoule protein content was determined spectrophotometrically. Ampoule integrity was assessed by determining residual moisture by the coulometric Karl-Fischer method (Mitsubishi CA100) and headspace oxygen content by frequency modulated spectroscopy using the Lighthouse FMS-760 Instrument (Lighthouse Instruments, LLC). No evidence of microbial contamination was found using the total viable count method.

Besides the lyophilized mAbs (coded A, B), six liquid mAb solutions (coded N to S), each containing 10 μg/ml of mAb in 20% normal human serum (First Link Ltd, UK) were included in the study. Brief characteristics of these antibodies are given in **Table 3**. Sample N was a mixture of mAbs INA29 and INA79 in equal ratio while O - S were individual mAbs (6, 11, 35), generously provided by collaborators based at Sanquin (Amsterdam, Netherlands), National Institute of Health Sciences, NIHS (Kangawa, Japan) or purchased from a commercial supplier (Biorad Laboratories Inc, US). A panel of 7 human serum samples (pools) from infliximab-treated patients with varying ADA levels as indicated in **Supplementary Table 1** (but none or undetectable levels of infliximab), sourced from a UK hospital and healthy control subjects (First Link Ltd, UK) were also included. Appropriate ethical approval was sought and materials anonymised for use. The samples were stored at -40°C until dispatch or use.

**Table 3:** Details of liquid monoclonal antibody preparations.

Sample code	Antibody origin	Clone/Other identifier	Isotype	Light chain	Binding Affinity KD (M)^1^	Reference
**N**	human PBMCs	INA29 + INA79 (1:1)	IgG1 + IgG4	κ	unknown	–
**O**	human B Cells	cl 1.4	isolated as IgG4, produced as IgG1	λ	2.59 E-10	(6, 10, 11)
**P**	human B Cells	cl 2.2	isolated as IgG4, produced as IgG1	κ	1.34 E-10	
**Q**	chimeric human-rat	cl I28-2G-IgG1	IgG1	κ	1.13 E-9	(35)
**R**	chimeric human-rat	cl I28-2G-IgG4	originally IgG1, produced as IgG4	κ	9.55 E-10	
**S**	Human (HuCAL phage display library)	HCA233/ AbD20436_hIgG1	IgG1	κ	1.2 E-10 (monovalent Fab)	–

N – mixture of the two ABIRISK antibodies (1:1); O, P – sourced from Sanquin, Netherlands; Q, R – NIHS, Japan; S - affinity matured anti-idiotypic antibody, commercialized for use in a bridging ELISA for measuring free drug or as a control or calibrator for human ADA bridging assays. ^1^Binding assessed using SPR (Biacore) for O – R, S – no information. All antibodies were determined to be neutralizing by the donors using different assays. O and P were tested in the TNF-sensitive WEHI-164 cell-based cytotoxicity assay; Q and R in the reporter gene assay using the GloResponse™ NF-κB-RE-luc2P HEK293 cell line (Promega); N using competitive ligand binding assay; S – not known.

### 2.2 Participants, study design and methods

As mentioned in the Introduction, a collaborative study for assessing the suitability of the infliximab ADA panel to serve as performance indicators for ADA assays was organized. The study also evaluated the feasibility of assigning an arbitrary unitage to the lyophilized mAbs to enable calibration of local standards and for assay harmonization.

Seventeen participants from 11 countries contributed data to this study. Participants represented contract research organizations (n=2), national control agencies (n=2), academic laboratories (n=3), commercial kit/therapeutic product manufacturers (n=4), clinical diagnostic centers/hospital laboratories (n=5) and a commercial supplier (n=1). For confidentiality, all participant data are blind coded with a randomized laboratory number (1–17) which is not related to the order of listing. In cases where the same laboratory returned two sets of data from two different methods, data was analyzed separately for each method as if from different laboratories and given a numerical code followed by a suffix such as 1a, 1b, 1c. Participants were encouraged to use their in-house qualified or validated methods and were sent a study-specific protocol with information on study aims and objectives, study samples with specific instructions on their storage and handling, reconstitution details (where appropriate), examples of suggested assay/plate layouts and a template for reporting of results. An independent statistical analysis of all data was centrally performed at MHRA.

Prior to the study launch, a survey was conducted which informed on the study design. As expected, a variety of assays (e.g., in-house assays, commercial kits) with varying sensitivity and assay range were in use. Differences in terms of the positive control/standard (polyclonal, monoclonal, human/animal species), its unitage (mass units, arbitrary units etc.), the use of quality control samples, sample treatment (e.g., dilution), sample diluent and the number of samples that could be positioned on a single plate were also noted. Majority of assays measured ‘free’ antibody except for assays in labs 6, 7, 9, 10 and 17 which measured free antibodies.

Feedback also indicated that participants could be categorised into two groups: those performing clinical monitoring as in hospital/diagnostic centre setting and assessing samples only at single dilutions routinely and those able to serially dilute samples to define a titre/endpoint. This led to designing a study protocol with certain aspects common to all but configured in such a way to provide the flexibility needed to accommodate differences which can occur in routine ADA testing.

Participants were sent 5 ampoules of samples A and B and adequate amounts of the liquid preparations as well as unknown serum samples for each assay type they intended to perform. Use of a freshly reconstituted lyophilized sample or a freshly thawed aliquot for each assay was recommended to ensure that samples were treated consistently for the study.

Participants were encouraged to test for binding and neutralizing activity of the samples using their own in-house qualified or validated methods (e.g., own proprietary kits, commercially purchased kits or methods developed in-house) and include routine controls and in-house reference standards (IH) where feasible. For dilution of in-house/kit standard or serum samples, use of matrix employed routinely was recommended.

Prior to performing the study assays, participants were advised to conduct a pilot assay and test all samples in parallel with in-house standard(s) and quality control (QC) samples to ensure optimal dose response curves and dilutions could be achieved for all samples (incl lyophilized mAbs) in the study runs. Although testing of clinical samples as per the procedure/dilution(s) used for routine testing was advised, non-clinical laboratories were urged to perform serial dilutions to achieve an assay endpoint where possible.

Participants were requested to report assay data for each tested sample based on their reporting practice e.g., qualitative (antibody +ve/-ve) or quantitative (e.g., titer or ADA concentration in mass/ml or arbitrary units/ml) relative to in-house/kit standards and if possible relative to mAbs coded A or B for each assay.

### 2.3 In-house evaluation of characteristics of the mAbs

Binding experiments employing the surface plasmon resonance (SPR) platform were performed on a Biacore T200 instrument, using a BioCAPture kit. Infliximab labelled with biotin at a challenge ratio of 1:1 was diluted to 45 μg/ml in running buffer (HBS-EP+ buffer: Hepes buffered saline with EDTA and surfactant P20) and captured on the CAP sensor chip by a 240-sec injection at a flow rate of 30μl/min. No biotin-infliximab was captured on the control flow cell. The mAbs were injected for 600 sec at a flow rate of 30μl/min, at concentrations of 20, 100, 500 and 1500ng/ml.

For binding affinity determination, single cycle kinetic experiments were conducted. Biotin labelled Infliximab (same as above) was diluted (1μg/ml in running buffer) and captured on the CAP sensor chip by a 120-sec injection at a flow rate of 30μl/min. The mAbs at 5, 20 80, 320 and 1280 nM were then injected for 120 sec at a flow rate of 30μl/min, followed by a dissociation phase of 600-3600 sec. The kinetics parameters (association and dissociation) of the injected mAbs were calculated using the 1:1 binding model (Biacore Evaluation ver.3.1 software, Cytiva).

For assessing ADAs in bridging ELISA, infliximab (1μg/ml in phosphate buffered saline, PBS, 100μl per well) was immobilized in 96-well plates (Thermo Fisher Scientific, UK) overnight at 4°C. Plates were washed with PBS-0.05%Tween20, blocked with casein buffer (Thermo Fisher Scientific, UK) for 1h at room temperature, washed again and samples at appropriate dilutions in PBS-0.5%BSA distributed into wells (100μl per well) and incubated for 2h at room temperature on a plate shaker. After washing, the secondary reagent, infliximab labelled with HRP at a challenge ratio 4 HRP:1 Ab (Lynx Rapid HRP Ab conjugation kit, Biorad) was added at 125ng/ml (100μl per well) and the plates incubated for 1.5h at room temperature on plate shaker. TMB peroxidase EIA substrate kit (Biorad) was used for detection (100μl per well), the reaction stopped after color development with 1M sulfuric acid (50μl per well) and the absorbance read at 450 nm using the Spectramax M5 plate reader. This method was also used for assessing the reactivity of the ADAs with different infliximab products e.g., Remicade, Remsima and Flixabi.

For assessing ADAs by ECL, samples at appropriate dilutions in PBS-0.5%BSA were distributed in the wells of dilution plates and an equal volume of a mastermix of ruthenium-labelled infliximab (MesoScale Discovery, MSD, Gaithersburg, USA) and biotin-labelled (EZ-Link Sulfo-NHS-LC-Biotin, Thermo Fisher Scientific, UK) infliximab each at 250 ng/ml (both labelled as per manufacturer’s instructions with labelling ratio of 10:1) was added. After incubation for 1.5h at room temperature on a plate shaker, the mixtures were transferred to pre-blocked MSD streptavidin (50μl per well) and incubated for 1h at room temperature on a plate shaker. The plates were washed with PBS-0.05%Tween20, read buffer 1x (MSD Read Buffer T with surfactant 4x) added (150μl per well) and the plates read using MSD Meso QuickPlex SQ120 instrument.

For the competitive ligand binding assay for assessing the neutralizing activity of the infliximab ADAs, samples at appropriate dilutions in PBS-0.5%BSA were added in wells of dilution plates and an equal volume of the master-mix of ruthenium-labelled TNF and biotin-labelled infliximab each at 5 ng/ml was added. After incubation for 1.5h at room temperature on a plate shaker, the mixtures were transferred to pre-blocked MSD streptavidin plates (50μl per well) and incubated for 1h at room temperature on a plate shaker. The plates were washed with PBS-0.05%Tween20, read buffer 1x (MSD Read Buffer T with surfactant) added (150μl per well) and the plates read using the MSD Meso QuickPlex SQ120 instrument.

For assessing the neutralizing activity of the infliximab ADAs in the cell-based neutralization assay, infliximab ADAs were diluted in assay medium (DMEM - Sigma #D5671, 10% heat-inactivated FBS, 50U/ml penicillin, 50μg/ml streptomycin, 100μg/ml normocin, 2mM L-glutamine). Serial dilutions (25μl per well) were incubated with infliximab at 40ng/ml (50μl per well) in 96-well cell culture plates for 1h in a humidified CO_2_ incubator. A fixed concentration of TNF was added (320IU/ml, 25μl per well) and the mixture incubated at 37°C for 1 h. HEK-Blue CD40L cells (InvivoGen, France) at a density of 5x10^5^cells/ml (100μl/well) were then added to the mixture and the plates incubated for 20-24h in the incubator. The production of SEAP was determined by adding 20μl of the cell supernatant to 180μl of QUANTI-Blue substrate (Invivogen, France) and incubating the plates for 2h at 37°C prior to measuring the absorbance at 620nm in the Spectramax M5 plate reader.

### 2.4 Statistical methods

The estimated activities of coded study samples were calculated relative to sample A, sample B or in-house (IH) reference standard. For the estimates calculated relative to samples A or B these samples were assigned a nominal content of 50 µg/ml. Estimates calculated relative to IH are reported in a variety of different units (µg/ml, AU/ml, titre etc). Data were centrally analysed at MHRA using a sigmoidal curve model or parallel line analysis with log transformed responses. All calculations were performed using the software program CombiStats, available from European directorate for Quality of Medicines, EDQM (36). Model fit was assessed visually, and non-parallelism was assessed by calculation of the ratio of fitted slopes for the test and reference samples under consideration. The samples were concluded to be non-parallel when the slope ratio was outside of the range 0.67 – 1.50. Results from valid individual assays were combined to generate unweighted geometric means (GM) for each laboratory and these laboratory means were used to calculate overall unweighted geometric mean estimates. Variability between assays and laboratories has been expressed using geometric coefficients of variation (GCV = {10*^s^*-1}×100% where *s* is the standard deviation of the log_10_ transformed estimates).

## 3 Results

The development of the panel involved multiple steps including selection of an optimal formulation, lyophilized reference standards, coordination of a multi-center study, sample testing by participants, data analysis and unitage assignment. The results leading to the recommendations to the WHO Expert Committee on Biological standardization (ECBS) and finally the establishment of the WHO Reference Panel for Infliximab ADA in Oct’22 are presented.

### 3.1 Antibody characteristics

**Figure 1:**
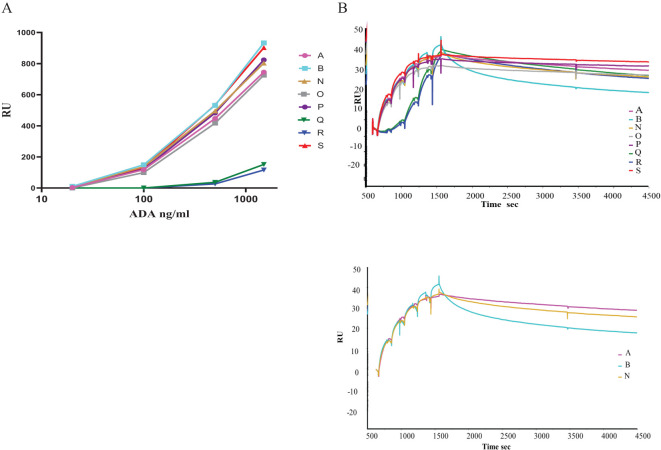
**(A)** Binding profile of the anti-infliximab mAbs, A and B (bulk material) and the liquid mAbs (N-S) as demonstrated by SPR. **(B)** Sensorgram generated from a representative single cycle kinetic experiment in which the anti-infliximab mAbs A and B (bulk material) and the liquid mAbs were comparatively assessed for affinity ranking (Top). The profile of the mAbs A, B (both bulk material) and mAb N (mixture of A and B) is also illustrated separately (Bottom).

**Figure 2:**
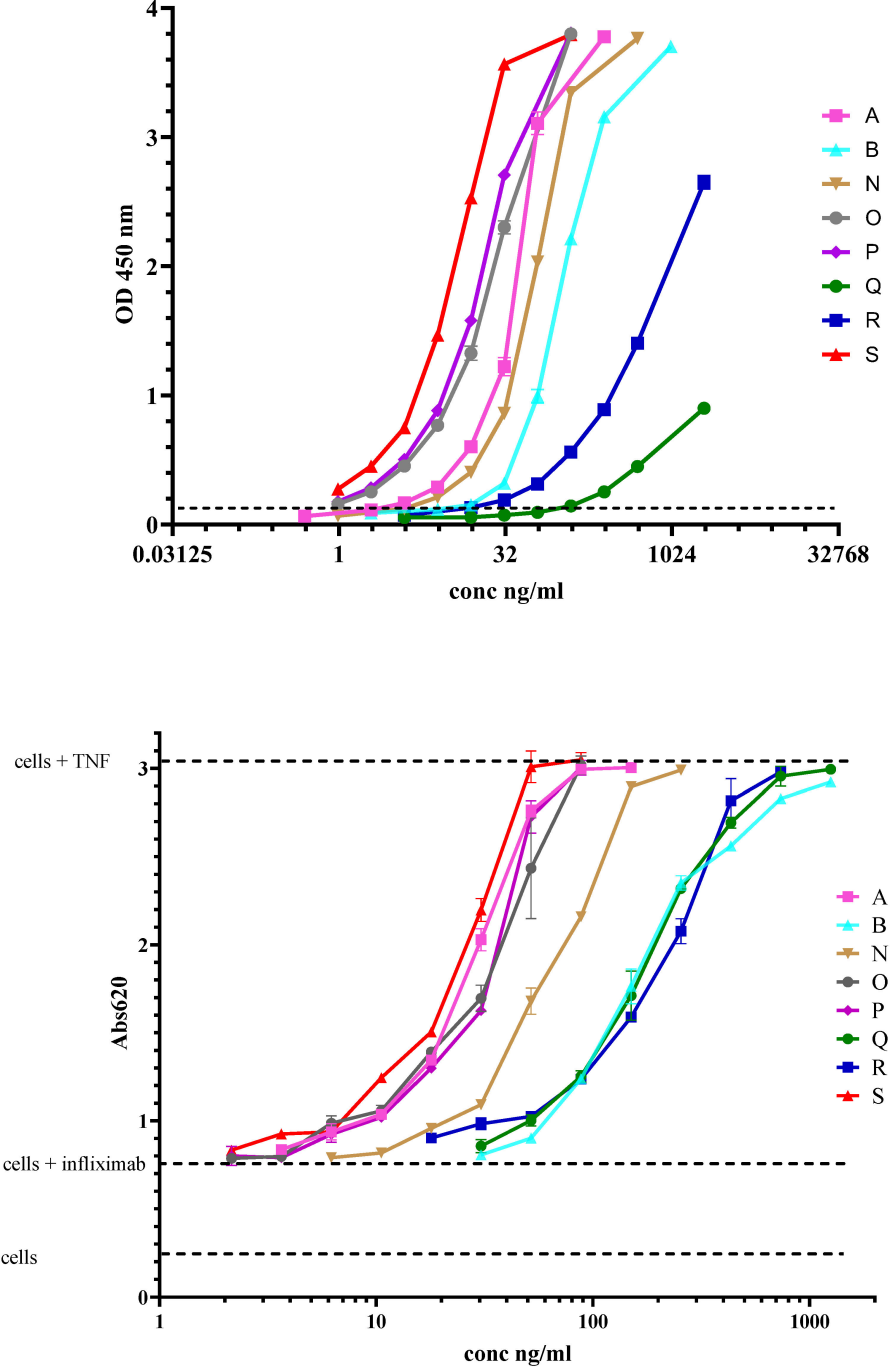
Data from a representative ELISA assay illustrating the binding profiles of the different infliximab ADAs (Top Panel). The hatched lines represent the cut-off level (average +1.645 StDev NC). Data from a cell-based bioassay showing the neutralizing profiles of the infliximab ADAs (Bottom Panel). The hatched lines represent the levels of SEAP detected with the assay controls (cells only, cell and infliximab, cells and TNF).

**Figure 3:**
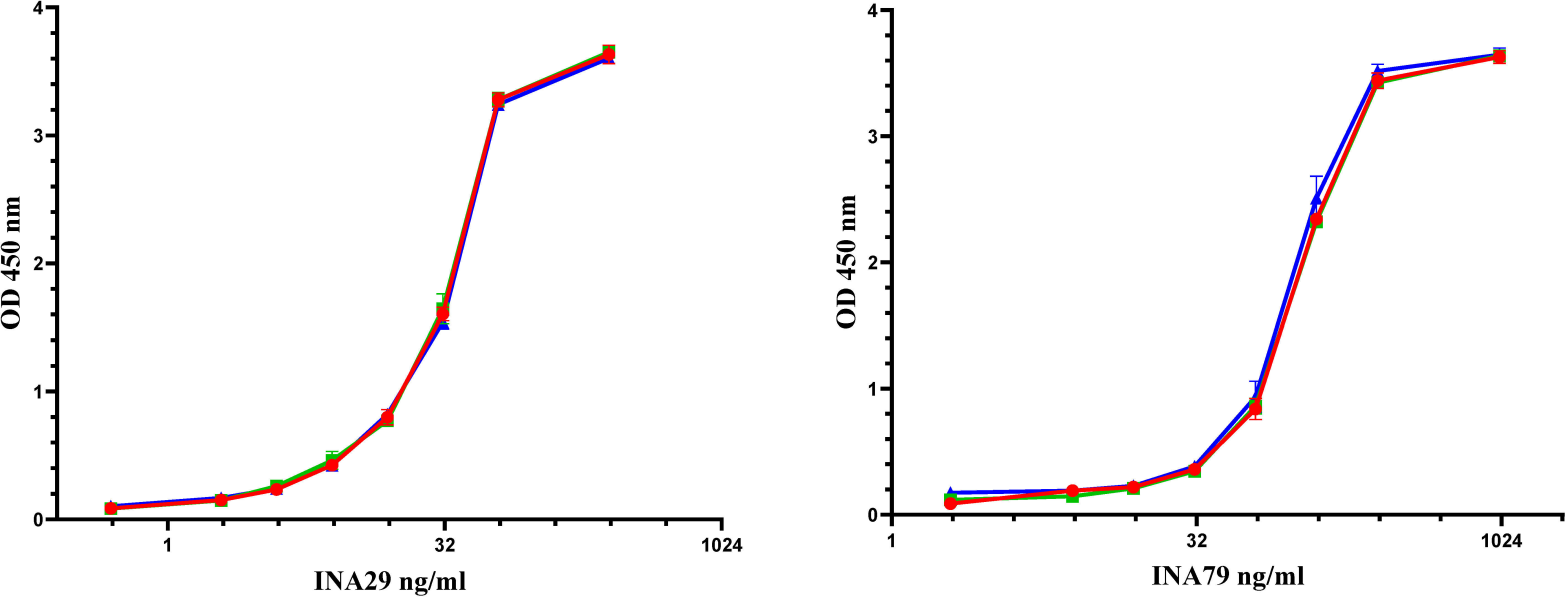
Detection of anti-infliximab mAbs A (INA 29) and B (INA 79) in a bridging ELISA using Remicade (red), Flixabi (green) or Remsima (blue).

Although information on the characteristics of the different antibodies included in the study was provided by different donors/supplier (**Tables 1**, **3**), a direct comparison of the binding characteristics was not possible due to varied methods used by the different laboratories. Binding activity of all the antibodies was therefore assessed at MHRA. For binding by SPR, biotinylated infliximab was captured on a CAP sensor chip and mAbs injected at different concentrations on the flow cells. The results of a typical experiment are shown in **Figure 1A**. The mAbs Q and R show significantly lower binding which is reflected by their lower affinity in comparison with the other mAbs which all showed high binding to infliximab. **Table 4** provides data on the association and dissociation of the different mAbs generated from single cycle kinetic experiments and indicates the ranking of the different mAbs in terms of affinities. The sensorgrams in **Figure 1B** illustrate the binding and dissociation profile of the different antibodies. Of note is the weak association of Q and R and the fast dissociation of the lyophilized antibody B. For all the mAbs, binding using a bridging ELISA and neutralization by the competitive ligand binding (non-cell-based) assay was also assessed (**Figure 2**). In addition, for the two lyophilized antibodies, reactivity with two different biosimilar products, Remsima and Flixabi was evaluated in an ELISA. The binding profile of the biosimilars was comparable with the innovator product, Remicade (**Figure 3**). This was confirmed using ECL assays for binding as well as for neutralization. As shown in the ELISA, comparable data was seen in both assays with Remicade and the two biosimilar products (data not shown).

**Table 4:** SPR binding data for the different antibodies in the study.

Antibody Code	ka (1/Ms)	kd (1/s)	KD (M)	Ranking Order (affinity)
**A**	6.338 E+5	6.391 E-5	1.008 E-10	4
**B**	7.288 E+5	2.111 E-4	2.896 E-10	6
**N**	5.482 E+5	9.605 E-5	1.752 E-10	5
**O**	7.168 E+5	3.999 E-5	5.580 E-11	3
**P**	6.689 E+5	2.274 E-5	3.400 E-11	2
**Q**	3.477 E+4	1.195 E-4	3.438 E-9	7
**R**	3.017 E+4	1.220 E-4	4.045 E-9	8
**S**	9.100 E+5	2.309 E-5	2.537 E-11	1

Results shown above were generated from a representative single cycle kinetic (SCK) experiment in which the infliximab ADA mAbs A and B (bulk material) and the liquid mAbs were comparatively assessed in a single run allowing for affinity ranking of the mAbs. The ranking order for the mAbs (A to S) in the 1^st^ column is based on high to low binding as shown in **Figure 1**.

### 3.2 Participant assays and data

For the study, all participants were requested to assess the activity of the different preparations and their in-house reference standards using their own in-house qualified methods. Details on the study design are provided in the materials and methods section. This practice allowed us to gain a valuable insight of the different types of assay systems that are currently in use in different laboratories and provided information on the behavior of the different ADA samples in these assay systems.

Participants contributed data from 22 different assay methods in total (**Table 5**). Most participants performed binding assays which are commonly used for ADA screening. Such binding assays varied from the simple ELISA and the commonly used electrochemiluminescence (ECL) or bead-based chemiluminescence assay, all using a bridging format to the radioimmunoassay type approach, termed antigen binding test (ABT) which is rarely used and the lateral flow (LF) assays which are increasingly offered as point-of-care devices. In clinical laboratories, most of the assays used were commercially available kits although in rare instances, in-house assays were also employed. For neutralization, laboratories performed competitive ligand binding assays as well as reporter-gene bioassays (including a commercial assay) using different cell-lines and read-outs. Reporting units varied among laboratories (**Table 5**), some using arbitrary units (AU/ml) while others using mass units (μg/ml) for reporting results relative to the in-house standard, or titres in rare cases and was consistent with the findings from the survey.

**Table 5:** Details of assays performed by study participants.

Lab	Assay (B/N) & Type	^1^C/IH	Positive Control/In-house standard			Units
			Nature	Use^2^	Assay range	
1a	B: Bridging ELISA	C	P	Cal	5-288^3^	AU/ml
2a		C	P	Cal	10-200	ng/ml
3		C	P	Cal	10-200	ng/ml
4		C	M	Cal	2.5-1000	ng/ml
5		C	H	Cal	20-640	ng/ml
6		C	H	Cal	5-450	AU/ml
7		C	H	Cal	20-185 ^4,5^	AU/ml
8		IH	P	Cal	2-250	ng/ml
9	B: Bridging ECL	IH	H^10^	Cal	9.8-1250	U/ml (ng/ml)^6^
10		IH	H^10^	AP	25-6000	ng/ml; titer
11		IH	H^11^	AP	2.4-625	ng/ml
12		IH	H	AP	3.12-200	ng/ml
13		IH	H^10^	AP	0.35-50	ng/ml; titer
17a		IH	P	AP	132-12000^4^	ng/ml; titer
1b	B: Lateral flow	C	ND	Cut-off	Pos/neg^7-9^	pos/neg (QCs: AU/ml)
15		C	H	Cal	200-15000	ng/ml
2b	B: Chemiluminescence	C	P	Cal	10-2000	ng/ml
14	B: Antigen binding test	IH	ADA	Cal	12-880	AU/ml
8b	N: Competitive ligand binding assay	IH	P	Cal	0.46-112	ng/ml
17b		IH	H	AP	19-240^4^	titer (QCs, range: ng/ml)
8c	N: Cell- based bioassay	IH	P	Cal	3.65-150	ng/ml
16		C	P	AP	Pos/neg	ng/ml

B, Binding assay; N, Neutralization assay; H, Human mAb; M, murine mAb cl MA-IFX10F9; ADA, ADA +ve human serum; P, Polyclonal antibody generated in rabbits was used as in-house reference standard in labs (1, 2, 3, 16, 17a) while lab 8 used sheep antibody. Labs 6 and 7 used human serum spiked with H while lab 12 used a cocktail of 4 human mAbs. Some labs sourced H from Biorad - HCA233^10^, HCA215^11^; ND, Not disclosed.

8b – ECL; 8c, 16 – bioassays; ^1^C or IH – commercial or in-house assay; Use^2^: Cal for assay calibration, AP for assessing assay suitability/performance; ^3^range can be extended to 1,440 or 28,800 with the additional recommended dilutions; ^4^based on in house QC (^5^patients pooled sera); ^6^results were provided in U/ml but range here in ng/ml; ^7^qualitative test but for this study, the assay provided values in ^8^titre or ^9^μg/ml. All assays measure free antibody except for assays in labs 6, 7, 9, 10, 17 which measure total antibody.

The liquid mAb samples N - S and serum samples 1-7 were tested by 16 participants, exceptions were laboratory 11 (sera not tested) and 7 (mAbs not tested). While the mAb samples were mainly tested using serial dilutions, serum samples were tested at a single dilution (n= 7 laboratories) and using serial dilutions (n= 9 laboratories). All samples were typically tested in at least 3 assays, with exceptions being laboratories 3 (liquid mAbs tested 2x), 9 (serum samples 2x), 10 (N 1x) and 17 (single assay for binding and neutralization).

Most participants used the mAb coded A as reference standard on each plate. B was tested in all labs but rarely used as the standard except in 4 labs. Data analysis indicated that B does not provide a dose-response in some assays (e.g., labs 6 and 7) and furthermore when binding activity was calculated relative to B, more variability was evident than with A (for the ELISA data at least). Consequently, statistical analysis and estimates were calculated relative to A.

### 3.3 Parallelism

Prior to estimating the activity in the samples, the parallelism of two samples tested at serial dilutions was assessed using the ratio of their fitted slopes as calculated by CombiStats with a value of 1.0 indicating perfect parallelism. For analyses using sample A as reference, the proportions of slope-ratios within various ranges are summarized in **Table 6** for the different samples and assay types.

Table 6Distribution of slope-ratios (relative to sample A) for the different samples and assay types based on participant data.

**Table:** 

ELISA							ECL						
Sample	Slope ratios vs A						Sample	Slope ratios vs A					
	% <0.67	% 0.67-0.80	% 0.80-1.25	% 1.25-1.50	% >1.50	n		% <0.67	% 0.67-0.80	% 0.80-1.25	% 1.25-1.50	% >1.50	n
B	8	0	80	12	0	25	B	0	0	100	0	0	12
N	0	0	88	0	12	25	N	0	0	100	0	0	10
O	17.6	0	82.4	0	0	17	O	0	21.4	57.1	14.3	7.1	14
P	0	0	94.1	5.9	0	17	P	0	21.4	57.1	14.3	7.1	14
Q	37.5	25	37.5	0	0	16	Q	50	14.3	35.7	0	0	14
R	52.9	17.6	29.4	0	0	17	R	28.6	7.1	64.3	0	0	14
S	0	12.5	87.5	0	0	16	S	0	7.7	69.2	7.7	15.4	13
2	0	33.3	66.7	0	0	6	2	0	0	100	0	0	2
3	33.3	0	66.7	0	0	9	3	40	20	40	0	0	5
4	0	0	100	0	0	9	4	40	20	40	0	0	5
5	16.7	0	83.3	0	0	6	5	0	0	100	0	0	3
6	0	0	100	0	0	6	6	0	0	66.7	33.3	0	3

**Table:** 

Neutralisation							Other						
Sample	Slope ratios vs A						Sample	Slope ratios vs A					
	% <0.67	% 0.67-0.80	% 0.80-1.25	% 1.25-1.50	% >1.50	n		% <0.67	% 0.67-0.80	% 0.80-1.25	% 1.25-1.50	% >1.50	n
B	22.2	44.4	33.3	0	0	9	B	0	0	88.9	11.1	0	9
N	0	0	100	0	0	9	N	0	0	88.9	11.1	0	9
O	0	0	100	0	0	7	O	22.2	0	77.8	0	0	9
P	0	14.3	85.7	0	0	7	P	0	0	88.9	11.1	0	9
Q	71.4	0	28.6	0	0	7	Q	25	0	75	0	0	4
R	57.1	42.9	0	0	0	7	R	20	20	60	0	0	5
S	0	0	100	0	0	7	S	0	0	88.9	11.1	0	9
2	42.9	42.9	14.3	0	0	7	2	100	0	0	0	0	2
3	0	57.1	42.9	0	0	7	3	22.2	44.4	22.2	0	11.1	9
4	0	28.6	71.4	0	0	7	4	0	11.1	66.7	11.1	11.1	9
5	42.9	28.6	28.6	0	0	7	5	0	0	100	0	0	2
6	16.7	16.7	66.7	0	0	6	6	0	0	100	0	0	2

**Table:** 

All methods						
Sample	Slope ratios vs A					
	% <0.67	% 0.67-0.80	% 0.80-1.25	% 1.25-1.50	% >1.50	n
B	7.3	7.3	78.2	7.3	0	55
N	0	0	92.5	1.9	5.7	53
O	10.6	6.4	76.6	4.3	2.1	47
P	0	8.5	80.9	8.5	2.1	47
Q	46.3	14.6	39	0	0	41
R	41.9	18.6	39.5	0	0	43
S	0	6.7	84.4	4.4	4.4	45
2	29.4	29.4	41.2	0	0	17
3	23.3	30	43.3	0	3.3	30
4	6.7	13.3	73.3	3.3	3.3	30
5	22.2	11.1	66.7	0	0	18
6	5.9	5.9	82.4	5.9	0	17

n indicates the total number of assays included for determination of parallelism; darker green shading indicates a higher percentage of slope ratios within the range shown, with better parallelism when darker shading is located in the central column (slopes ratios in range 0.80 – 1.25).

Analysis of the slope ratios relative to A clearly showed that for the liquid mAb samples, the slopes were within a range of 0.67-1.50 in most assays. The degree of parallelism varied depending on the mAb, the assay platform and the laboratory. Good parallelism (100% of results within slope ratio of 0.80-1.25) was seen with samples N in ECL and N, O and S in neutralization assays. In ELISAs, over 80% of the results for four liquid mAbs (N, O, P, S) were within 0.80-1.25, the notable exceptions were mAbs Q and R, which behaved differently and distinctly demonstrated non-parallelism. A similar situation was also observed for these mAbs in neutralization assays. In other assays, for example, lateral flow, radioimmunoassay, chemiluminescence, three mAbs showed 80% of results within 0.80-1.25 whereas in ECL, this was only evident with one mAb (**Table 6**). B showed good parallelism in the ECL assay but non-parallelism was evident in the neutralization assay. As for serum samples, two of five sera (coded 2 and 5 in ECL, 4 and 6 in ELISA, 5 and 6 in other assays) showed 100% parallelism (within slope ratio range of 0.80-1.25) in the different binding assays but all were non-parallel in the neutralization assays (**Table 6**).

Based on varying degree of parallelism, for comparative analysis of the calculated concentrations and determination of the GM and %GCV, all estimates calculated from cases where the slope-ratio was within 0.67-1.50 were used. All other cases were excluded as showing an unacceptable level of non-parallelism.

### 3.4 Estimates of activity calculated relative to A and IH standard

Estimates from binding (n=18) and neutralization assays (n=4) for the mAb and serum samples calculated relative to sample A or relative to the IH standards (where available) are shown in **Tables 7A, B** and briefly summarized below. Detailed results of individual labs are provided (**Supplementary Table 2**); ELISA data is given in **Table 8**. A few laboratories reported binding activity in AU/ml relative to the in-house/kit standard and neutralizing activity in titers, but comparisons were only possible for data where laboratories reported assay results in μg/ml. Additionally, a comparison of data from drug-sensitive and drug-tolerant assays was not possible as only five laboratories (labs 6, 7, 9, 10, 17) performed total ADA assays, 3 of which expressed results in either U/ml or AU/ml and one laboratory tested only some of the samples.

**Table 7A:** Summary of geometric mean (GM) estimates and geometric coefficients of variation (GCV) for ELISA and ECL assays.

ELISA							ECL					
Sample	Estimates vs A (μg/ml)			Estimates vs IH			Estimates vs A (μg/ml)			Estimates vs IH		
	Range	GM	GCV	Range	GM	GCV	Range	GM	GCV	Range	GM	GCV
mAbs												
**A**				17.80 - 1129.34	92.83	613%				6.55 - 246.05	32.00	303%
**P**	16.91 - 37.39	21.90	37%	7.54 - 405.55	46.06	459%	8.47 - 44.66	23.52	100%	3.68 - 183.97	14.92	340%
**O**	10.64 - 30.3	16.61	46%	6.89 - 326.16	38.34	453%	8.06 - 57.74	23.26	111%	2.53 - 172.04	14.78	398%
**S**	12.05 - 98.8	14.94	26%	9.54 - 374.52	37.91	451%	29.31 - 47.04	38.12	20%	3.87 - 220.85	22.98	355%
**B**	0.15 - 14.33	7.36	97%	0.90 - 139.59	18.07	982%	25.31 - 105.87	53.86	84%	3.32 - 246.84	26.81	545%
**N**	3.51 - 6.82	5.16	29%	1.25 - 128.06	13.49	791%	6.87 - 14.84	10.09	36%	0.89 - 42.49	7.56	410%
**R**	0.21 - 1.19	0.37	172%	0.13 - 4.84	0.70	391%	0.63 - 10.33	3.62	222%	0.25 - 50.28	2.27	493%
**Q**	0.06 - 0.61	0.10	100%	0.01 - 1.34	0.16	1044%	0.49 - 4.49	1.97	162%	0.34 - 12.77	1.49	258%
Sera												
**4**	3.51 - 10.47	5.14	64%	1.74 - 114.02	10.55	759%	5.02 - 22.57	10.44	89%	1.63 - 53.83	4.83	301%
**3**	1.34 - 8.34	3.73	131%	0.70 - 91.32	6.71	1063%	1.95 - 19.17	7.59	163%	1.24 - 45.72	3.82	338%
**6**	0.09 - 2.43	0.17	77%	0.07 - 3.71	0.39	678%	0.35 - 3.89	0.81	193%	0.06 - 2.46	0.26	497%
**5**	0.06 - 1.80	0.12	92%	0.05 - 2.62	0.29	633%	0.26 - 2.01	0.56	144%	0.05 - 1.27	0.19	428%
**2**	0.02 - 1.03	0.04	107%	0.02 - 0.95	0.11	478%	0.11 - 0.41	0.22	79%	0.03 - 0.95	0.13	300%

Samples 1, 7 were negative so not included. Results based only on labs whose results are reported in μg/ml.

**Table 7B:** Summary of geometric mean (GM) estimates and geometric coefficients of variation (GCV) obtained for the different assays (except for assays where data was limited, shaded blue) when A is used as the common standard.

Sample	ELISA		ECL		LF		CLIA	ABT	Neutralisation	
	GM	GCV	GM	GCV	Lab 1b	Lab15	Lab 2b	Lab 14	GM	GCV
mAbs										
**A**										
**P**	21.90	37%	23.52	100%	12.60	14.70	12.98	14.96	7.63	97%
**O**	16.61	46%	23.26	111%	7.94	12.40	10.35	17.60	9.79	29%
**S**	14.94	26%	38.12	20%	12.60	9.57	13.60	20.02	13.44	50%
**B**	7.36	97%	53.86	84%	15.75	43.71	11.87	8.04	14.18	137%
**N**	5.16	29%	10.09	36%	7.94	8.52	6.03	10.13	5.93	18%
**R**	0.37	172%	3.62	222%	Pos	0.4	0.04	1.44	1.69	
**Q**	0.10	100%	1.97	162%	Pos	0.2	0.02	1.27	1.48	
Sera										
**4**	5.14	64%	10.44	89%	6.20	8.97	3.37	19.19	11.79	21%
**3**	3.73	131%	7.59	163%	6.20	11.32	2.78	27.34	18.61	20%
**6**	0.17	77%	0.81	193%	0.16	0.2	0.12	0.44	0.26	16%
**5**	0.12	92%	0.56	144%	0.08	0.2	0.08	0.32	0.17	27%
**2**	0.04	107%	0.22	79%	0.08	0.2	0.03	0.61	0.42	

Samples 1, 7 were negative so are not included.

Table 8ELISA data for mAb preparations and serum samples. Geometric mean estimates and Geometric coefficients of variation (GCV) calculated vs sample A (μg/ml) (top panel) or vs IH/kit standards (bottom panel).

**Table:** 

Sample	Lab								Range	GM	GCV	GM*	GCV*
	1a	2a	3	4	5	6*	7*	8a					
mAbs													
**A**	50.00	50.00	50.00	50.00	50.00	50.00	50.00	50.00					
**P**	21.92	19.16	18.98	n/t	16.91	17.73	N/T	37.39	16.91 - 37.39	21.14	34%	21.90	37%
**O**	10.64	15.41	16.48	n/t	15.44	NP	N/T	30.30	10.64 - 30.3	16.61	46%	16.61	46%
**S**	12.05	16.18	12.46	n/t	21.38	98.80	N/T	14.34	12.05 - 98.8	20.47	122%	14.94	26%
**B**	9.89	6.60	9.48	2.43	N/T	0.15	NP	14.33	0.15 - 14.33	3.84	449%	7.36	97%
**N**	5.30	6.05	4.77	3.51	N/T	4.34	NP	6.82	3.51 - 6.82	5.02	27%	5.16	29%
**R**	NP	0.21	0.21	n/t	NP	NP	N/T	1.19	0.21 - 1.19	0.37	172%	0.37	172%
**Q**	NP	0.06	0.08	n/t	Neg	0.61	N/T	0.22	0.06 - 0.61	0.15	192%	0.10	100%
sera													
**4**	3.51	4.85	Pos	Pos	3.90	5.44	4.68	10.47	3.51 - 10.47	5.11	47%	5.14	64%
**3**	NP	3.94	Pos	Pos	1.57	1.34	4.39	8.34	1.34 - 8.34	3.14	114%	3.73	131%
**6**	0.09	0.16	Pos	0.19	0.12	0.26	2.43	0.40	0.09 - 2.43	0.26	200%	0.17	77%
**5**	0.06	0.11	Pos	0.15	0.08	0.18	1.80	0.33	0.06 - 1.80	0.19	212%	0.12	92%
**2**	0.02	0.04	Pos	0.04	Neg	0.11	1.03	0.11	0.02 - 1.03	0.09	301%	0.04	107%
**1**	Neg	Neg	Neg	Neg	Neg	Neg	Neg	Neg					
**7**	Neg	Neg	Neg	Neg	Neg	Neg	Neg	Neg

**Table:** 

Sample	Lab								Range**	GM**	GCV**
	1a	2a	3	4	5	6*	7*	8a			
**mAbs**											
**A**	31015.72	1129.34	534.44	17.80	22.30	78139.70	5335.95	28.78	17.80 - 1129.34	92.83	613%
**P**	12685.81	405.55	199.27	16.28	7.54	31268.3	n/t	20.91	7.54 - 405.55	46.06	459%
**O**	6155.22	326.12	173.04	12.58	6.89	48636.1	n/t	16.94	6.89 - 326.16	38.34	453%
**S**	7139.31	374.52	144.01	16.77	9.54	189131.6	n/t	9.08	9.54 - 374.52	37.91	451%
**B**	6137.33	139.59	107.28	0.90	n/t	287.60	36.93	7.87	0.90 - 139.59	18.07	982%
**N**	3287.96	128.06	55.19	1.25	n/t	7636.7	443.53	3.75	1.25 - 128.06	13.49	791%
**R**	12.34	4.84	2.20	0.13	0.15	385.4	n/t	0.75	0.13 - 4.84	0.70	391%
**Q**	Neg	1.34	0.80	0.01	Neg	1116.0	n/t	0.11	0.01 - 1.34	0.16	1044%
sera											
**4**	2335.40	114.02	>0.2	>1.0	1.74	>450	>200	5.91	1.74 - 114.02	10.55	759%
**3**	1700.59	91.32	>0.2	>1.0	0.70	>450	>200	4.71	0.70 - 91.32	6.71	1063%
**6**	59.54	3.71	>0.2	0.07	Neg	>450	225.66	0.23	0.07 - 3.71	0.39	678%
**5**	39.41	2.62	>0.2	0.05	Neg	343.10	164.44	0.18	0.05 - 2.62	0.29	633%
**2**	12.65	0.95	0.19	0.02	Neg	228.90	91.07	0.06	0.02 - 0.95	0.11	478%
**1**	Neg	Neg	Neg	Neg	Neg	Neg	Neg	Neg			
**7**	Neg	Neg	Neg	Neg	Neg	Neg	Neg	Neg			
**Units**	AU/ml	μg/ml	μg/ml	μg/ml	μg/ml	AU/ml	AU/ml	μg/ml			

NP, Non-parallel to standard (grey); N/T, Sample not tested by lab (dark blue); n/t, Sample not tested on same plates as standard (light blue); Neg, Sample reported as negative or below assay lower quantitation limit (orange); Pos, Sample reported as positive (green) but no quantitative estimate available (e.g., above assay upper quantitation at dilution(s) tested) relative to A; *GM and GCV also calculated with exclusion of these labs; **labs whose results are reported in μg/ml;

Samples are ranked based on the GM obtained for the ELISA vs IH standard (high to low).

#### 3.4.1 mAb preparations

All mAb preparations tested positive for ADA in the different assay platforms employed in the study. Despite use of the same assay type, differences were apparent among the assays in different labs. All mAbs showed reactivity in the different ECL assays (n=6), LF assays (n=2), CLIA (n=1), ABT (n=1) and the neutralization assays (n=4). However, disparity in recognition was evident among the bridging ELISAs. While six of eight bridging ELISAs (75%) detected ADA present in all the mAbs, the ELISAs in two labs (coded 1a and 5) failed to detect antibody Q (**Table 8**). For lab 1a, the negative result was noted relative to the in-house standard while for lab 5, the ADA was missed regardless of the standard used for quantitation. In total, 20 of 22 assays (91%) included in the study detected all samples.

**Figure 4:**
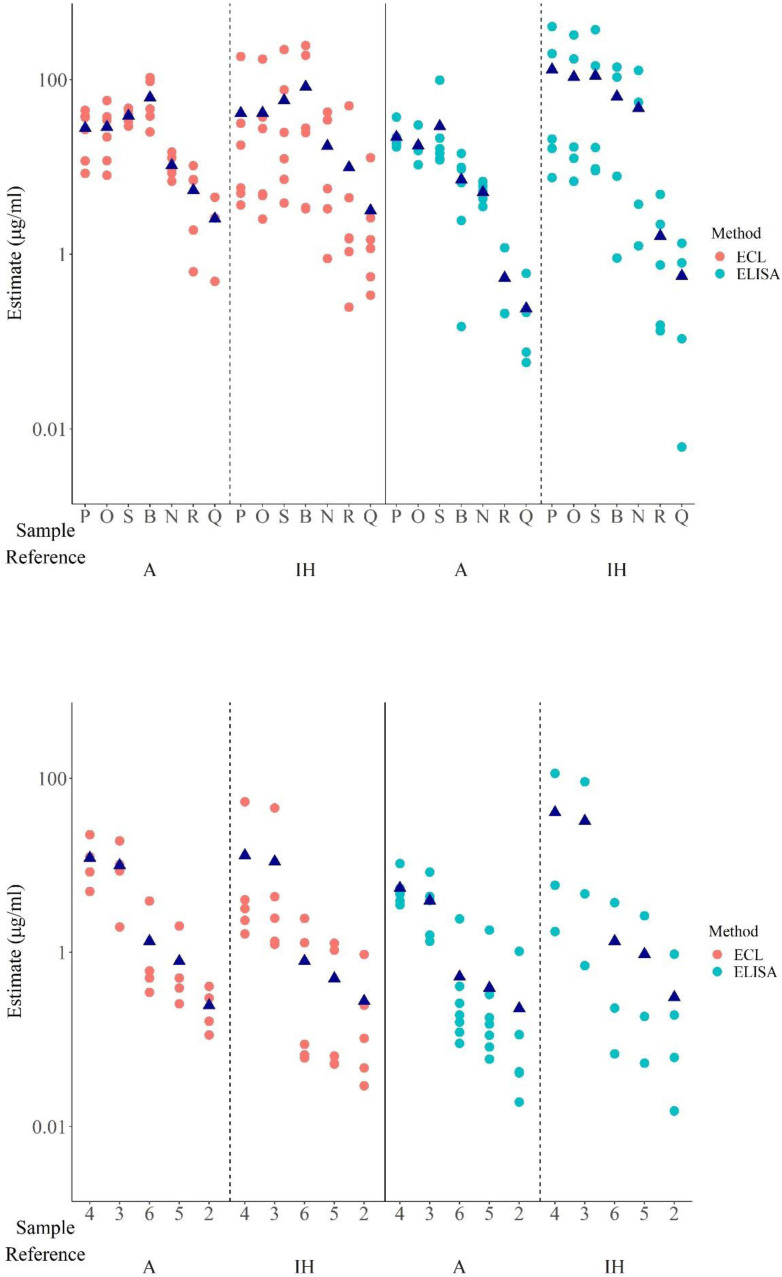
Scatter plot showing geometric mean estimates obtained in different ELISAs and ECL assays for monoclonal antibodies (Top panel) and serum samples (bottom panel) relative to A and in-house standard (IH). For IH, estimates from labs which provided data in ‘μg’ included. Dark blue triangles indicate geometric mean values for each sample.

**Figure 5:**
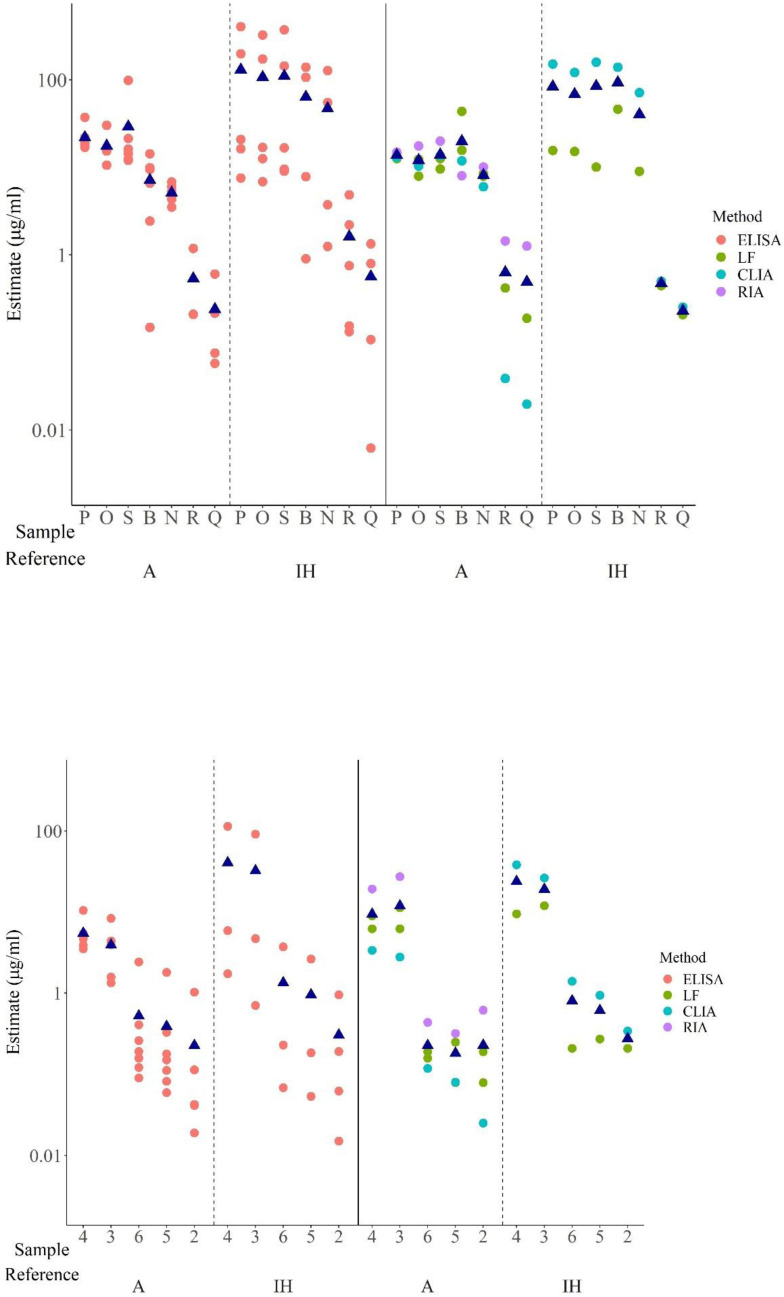
Scatter plot showing geometric mean estimates obtained in different assays for different mAbs (top panel) and serum samples (bottom panel) relative to A and in-house standard (IH). For IH, estimates from labs which provided data in ‘μg’ included. Dark blue triangles indicate geometric mean values for each sample.

Evidence indicated that the immunoreactivity of each mAb preparation varied not only among different assay platforms but also between laboratories employing the same assay platform. Such differential recognition led to wide differences in estimates of ADA levels among different labs when calculated relative to the in-house/kit standard, particularly for ELISAs (**Table 8**). Disparate ADA levels were also observed with ECL assays but the variation in ADA levels was highest among the different ELISAs in comparison with ECL assays (**Figure 4**). However, when A was used as the common standard, the variability in levels of ADA detected was considerably reduced despite the dissimilar estimates obtained in different assays for each mAb. For example, in ELISAs, relative to the in-house standard, sample P varied from 7.5 – 405.6 μg/ml whereas in ECL assays, values were 3.7 – 184.0 μg/ml but when calculated relative to A, the range varied from 17.7 – 37.4 μg/ml and 8.5 – 44.7 μg/ml in ELISAs and ECL assays respectively (**Table 7B**, **Figure 4**). As demonstrated in **Table 8**, the values from ELISA data of labs 1a, 2a, 3, 5 were quite consistent for all mAbs indicating harmonization among laboratories when A was used (**Figure 4**), which in certain cases, extends to similar values seen across different platforms (CLIA from lab 2b, ABT from lab 14, LF assays from 1b and 15, see **Table 7B**, **Figure 5**).

The variability between assays and laboratories has been expressed using geometric coefficients of variation (%GCV). The analysis of the %GCV by assay type showed that for both ELISA and ECL assays, the %GCV was large, amounting to a value as high as approximately 1000% and 545% respectively for estimates of ADA levels calculated using IH standard but was profoundly reduced relative to A as shown in **Table 7A**. For instance, in ELISAs, there was relatively good agreement between estimates (n=6) for P, O, S, B, N with %GCV ranging from 26% – 97% vs A (if results of participant 6 are excluded) in comparison with a GCV range of 451 – 982% relative to in-house standards although the %GCV vs A was still very large for some antibodies. This improvement in %GCV also applied to samples R and Q which demonstrated weak binding and therefore any numerical difference in the binding (in OD) of these mAbs in the different ELISAs and the subsequent calculation of concentration resulted in high variability and high impact as evidenced by the highest %GCV (391% and 1044% vs in-house which decreased to 172% and 100%) for these samples.

For ECL assays (n=6), the %GCV for all the mAbs vs A ranged from 20% to 222% (or 22% to 112% if data from lab 17 are excluded on the basis that only one assay was performed) which was a significant improvement from the %GCV range of 258 – 545% (or 200 – 483% if lab 17 data are excluded) relative to in-house standards (**Table 7A**). No %GCV could be calculated for the other assays (LF, ABT, CLIA) as there was data from only one or two laboratories, however, the range of concentrations detected for the different liquid mAbs were narrower when calculated vs A (**Table 7B**) which again indicated the advantage of using a common standard.

All mAb preparations showed neutralization activity. Surprisingly, for each of the mAb preparations, not much variation in levels was evident among the four neutralization assays which included both cell-based reporter gene and non-cell based competitive ligand binding assays (n=2 in each case) regardless of the standard used to calculate GM estimates for the samples. Importantly, mAb Q which displayed low or barely detectable binding activity and was even missed in the binding assays in some cases (ELISA of laboratories 1a, 5) was found to have neutralizing activity.

The ranking order of binding activity of the mAbs from high to low based on the geometric mean estimates from ELISAs calculated vs IH/kit standard is illustrated in **Table 7**. Although the exact ranking order varied between assays and the assay type, the mAbs coded P, O and S generally showed high/strong binding in all assays while N was identified as a moderate/low binder and Q and R as very low binders in most assays. Only in the ECL assay of lab 17, the ranking order was vastly different to the other labs with N showing higher binding than O and P although Q and R still ranked the lowest. Inclusion of the mAb B into the group altered the ranking order of the mAbs to some extent because of the differential binding of B in the different assay platforms. In ELISAs, B showed similar binding as N but lower than P, O and S and ranked higher than Q and R while in ECL and LF assays, it exhibited similar behavior as the other high binding mAbs e.g., P, S, O. In LF assays, B was the highest-ranking sample (excluding sample A) irrespective of the standard (A or the IH/kit standard) used for calculating the activity. In contrast, sample S was ranked as the highest in the ABT and CLIA assays. In most assays, N appears to be the lowest ranked sample, if Q and R are considered an exception and excluded based on data from all binding and neutralization assays.

#### 3.4.2 Serum samples

Two of the seven serum samples, coded 1 and 7, were identified as negative in all 21 assays where tested. For the residual positive samples, however, discrepancies were noted. While most assays/laboratories were able to identify these five samples as positive regardless of the standard used for calculating binding activity, laboratory 5 was a notable exception. In the ELISA from laboratory 5, the positivity of some of the serum samples varied depending on the standard used. Consequently, samples 5 and 6 were detected as positive, albeit at low levels, relative to Sample A but were found to be negative relative to the in-house standard. However, other samples e.g., the moderate/high positive samples, 3 and 4 were detected as positive while sample 2 was negative and not impacted by the standard. Just like for mAbs, levels varied considerably for the serum samples among assays/laboratories despite use of the same platform. A wider range of ADA levels and high variation was evident in the case of both ELISAs and ECL assays when the in -house standard was used as opposed to sample A (**Tables 7A, B**, **Figure 4**, **Supplementary Table 2**). The %GCV for both these assay types, particularly the ELISAs was considerably reduced when A was used as a common standard for determining ADA levels in the serum samples (**Tables 7A**, **8**).

All samples positive in the binding assays showed neutralizing activity. Although data are limited, estimates for neutralizing activity were generally consistent among the different assays/labs and across use of different standards. The values for %GCV for the serum samples in neutralization assays were lower when using A as reference material (16 – 27%) instead of an IH/kit control (56 – 120%).

In **Table 7**, the ranking order of binding activity of the samples from high to low based on the GM estimates from ELISAs calculated vs IH/kit standard is shown. The ranking of the samples was very similar across both ELISA and ECL assays for estimates relative to IH standard or sample A although in some cases, there was a slight variation in the exact ranking order. Generally, samples 3 and 4 were ranked in the top two with sample 4 ranked first among most assays although in individual ECL assays performed by labs (10 and 13), sample 3 showed a slightly higher binding than sample 4. The same was seen for labs 14 (ABT assay) and 15 (LF assay). Unusually, the ECL assay from lab 12 identified sample 3 below samples 6 and 5 regardless of the standard. For most participants, sample 2 was the one with lowest binding activity. The exceptions were labs 10 (vs IH/kit) and lab 14 (vs either standard), for which the observed binding activity is higher than the activity of samples 5 and 6. The ranking of the serum samples for lab 2b (CLIA assay) was the same as the one determined for the ELISA and ECL assays.

For neutralization activity, ranking is consistent between assays and across standards. In all assays, sample 3 showed the highest neutralization while sample 5, the lowest neutralizing activity. For the neutralization assay of lab 17 where results vs IH/kit standard were not included due to non-parallelism, samples 3 and 4 had the same titer (512). Serum sample 2, which showed the lowest binding activity in most assays (except for ABT assay of lab 14 and ECL assay of lab 10 vs IH), displayed higher neutralizing activity than the serum samples 5 and 6 in all neutralization assays, when activity was calculated vs IH/kit standard or vs A.

### 3.5 Stability testing

As per WHO guidelines, stability assessment of the lyophilized mAbs was also carried out. For the accelerated thermal degradation study, both 19/234 (A) and 19/232 (B) were stored at elevated temperatures (4°C, 20°C, 37°C and 45°C) for up to 22 and 23 months respectively and tested in-house with ampoules stored at -70°C and at the recommended storage temperature of -20°C using an ELISA and the HEKBlue-CD40L reporter gene neutralization assay. No loss in activity of both mAbs was evident following storage at elevated temperatures up to 20°C (**Supplementary Table 4**). Furthermore, both preparations were also found to be stable with repeated freeze-thaw cycles (up to 4) (**Supplementary Table 5**). These results clearly indicated that the lyophilized mAbs 19/234 (A) and 19/232 (B) are stable and suitable for use.

## 4 Discussion

Immunogenicity testing of a biotherapeutic is a regulatory expectation both for approval and for pharmacovigilance purposes. It is widely recognized that immunogenicity data is highly dependent on the assays used. As each assay platform has its own strengths and weaknesses, choice of the most appropriate assay platform/formats prior to validation and use in clinical studies is critical for generating reliable data from immunogenicity testing. A suitable positive control for assay validation and for monitoring assay performance is also important. ADA assays typically use a “surrogate” as positive control which is mainly of animal origin although in rare cases, purified ADA from human serum may be used (post-licensure clinical studies). Polyclonal antibodies, being heterogeneous in nature are ideal for representing the immune response and are often chosen for use in assay development and validation. However, potential reproducibility issues associated with replacement batches can impact assay performance precluding their long-term use. Positive controls using mAbs therefore are often favored for assay performance monitoring and for life-cycle management.

For biotherapeutics, the high through-put bridging format assays, particularly the ECL-based assays (37) continue to be the assay of choice for most regulatory submissions while the ELISA (38) is dominant for commercial kits. Radio-immunoassays (RIA) are generally less common (39, 40). However, they are sometimes the preferred option in clinical laboratories because of some special assay characteristics (e.g., sensitivity, ability to detect the IgG4 isotype, less target and drug interference) as compared with bridging assays which can suffer from target and drug interference and may not detect IgG4 antibodies (16, 19). This is also true for infliximab (41–47) where drug-tolerant ADA formats (measure total ADA) with improved ability to detect ADA have been developed but drug-sensitive (only unbound ADA measured) assays continue to be used based on their ability to detect clinically relevant ADA (20). Use of homogeneous mobility shift assay (HMSA) (48, 49) and SPR (50) has also been explored. Evidence has shown greater potential of ADA detection by SPR in patient sera considered ADA-negative by ELISA (51). For clinical monitoring purposes, the point of care rapid lateral flow assays have gained considerable momentum (46, 47, 52). For neutralizing capacity, several options exist - most favored are the reporter gene assays (53) and competitive ligand binding assays (54, 55) but other platforms such as LC-MS/MS have also been investigated (56).

For some infliximab treated patients, immunogenicity is a concern as responsive patients gradually become treatment resistant. Routine clinical monitoring of drug and/or ADA levels where appropriate continues to be extensively debated largely due to lack of strong evidence of the benefits of TDM (efficacy, cost-effectiveness) as well as the lack of standardization of analytical methods and/or reference standards for measuring ADA (and the TNF inhibitor itself). Availability of many commercial kits which differ in sensitivity, cut-off criteria (for distinguishing ADA positive samples), reporting units and other aspects have also added to the complexity. Consequently, there has been a strong demand for standardization of these methods by clinicians (7, 19, 21, 26, 27) and the National Institute for Clinical excellence (NICE, UK). In parallel, EULAR has recommended reactive TDM for management of inflammatory rheumatic and musculoskeletal diseases (57) and evidence on the predictive nature of TDM on patient outcome in different indications is now emerging (13–15, 58).

In this study, we evaluated two monoclonal antibodies which are representative of the antibody repertoire in infliximab treated patients for their suitability to serve as positive controls for infliximab ADA assays. From the characteristics described in **Tables 1** and **4** and the behavior elicited by antibodies A and B, it is evident that both these antibodies are quite distinct not only in terms of their isotype but also in their affinity and their dissociation profile. Antibody A is a high affinity IgG1 antibody which shows strong binding in the different assay types while B, in contrast, is an IgG4 antibody (stabilized mutein) with variable binding activity and a fast dissociation rate (**Figure 1**). This unique property led to the differential recognition of B in the assays in which it was tested and translated into higher estimates for ADA in the ECL assay in comparison with the ELISA where it is likely more susceptible to the washing steps incorporated in the procedure. Based on the properties of A and B and the study data, it became apparent that A could serve as a reference standard for assay calibration while B which exhibited variable binding, could be used to assess the ability of the assay to detect rapidly dissociating antibodies.

Despite the caveats associated with this collaborative study, several points were noted. Firstly, analysis of study data revealed problems with parallelism. This was largely seen with the mAbs Q and R which behave differently in comparison with other mAbs (**Tables 6**, **8**, **Figure 1**, **Supplementary Table 2**) and the serum samples. The non-parallelism noted with sera was not unexpected. It is well recognised and reported previously (27) since polyclonal antibodies are a heterogeneous mixture of antibodies of varied affinities, avidities and isotypes. Secondly, the results were expressed as μg/ml or as AU/ml relative to the IH/kit standards. The coexistence of several reporting methods precluded the possibility of a comparison of the results from all the different laboratories. Thirdly, even after segregation by assay type and reporting units, the study data clearly showed that the ability to detect and quantify ADA differed profoundly between assays/assay platforms when the IH standard was used and led to either lack of ADA detection in some cases to very wide estimates of ADA levels in others among the various samples tested. For example, the reported concentrations for Sample A in ELISAs relative to IH/kit standards varied from 17.80 to 1129.34 μg/ml. Lastly, the failure to detect ADA in some samples which was noted in some assays despite use of the same assay platform in various laboratories indicates an inherent problem with the particular assay employed in a specific laboratory rather than the assay format. Such discrepant observations were not unexpected given that the nature of the positive controls employed (and used for deriving ADA levels in some cases) in the different ADA assays varied from either a human or murine monoclonal antibody or a cocktail of human monoclonal antibodies to purified polyclonal animal sera or ADA positive human sera. Importantly, the findings albeit with only a limited number of laboratories, highlighting discrepancies in ADA detection and/or levels that were mostly seen with ELISAs (which are widely used for clinical monitoring) emphasized the need for assay harmonization. In some cases, the need for further assay optimization and validation to enable detection of all ADAs was also observed. In addition, it was interesting to note that even laboratories using the same commercial kit for measuring anti-infliximab antibodies (labs 6 and 7, 2a and 3) reported disparate results highlighting the importance of batch-to-batch consistency between kits/reagents, use of independent in-house standards and the influence of assay design and execution, consistent analysis and interpretation of data.

The use of a ‘common standard’, sample A did not allow the results from the different laboratories to flawlessly align. This is possibly due to differences in the vast array of assays/protocols used (e.g., minimal required dilution), and their capability in terms of antigen-antibody complex formation, drug tolerance, choice of reagents and/or conjugation procedures where relevant, the assay designs (e.g., inappropriate choice of dilutions range), assay execution and data analysis (e.g., cut-off values as indicated in **Supplementary Table 3**). Nevertheless, the results of this study undoubtedly showed less inter-laboratory variation and improvement towards consistent estimates when results were reported relative to sample A as opposed to the in-house/kit standards. Furthermore, the use of A allowed ADA detection in cases where ADA was missed and is particularly applicable to ELISAs performed in labs 5 and 1a. These labs failed to detect ADA in some samples (e.g., mAb Q missed by labs 1a and 5; serum samples 5, 6 missed by lab 5) when IH standards were used. Of importance is that all the ADAs missed had neutralizing activity. Our findings indicating that the use of a common standard can enable ADA detection and potentially harmonize results across assays/platforms and laboratories has important implications for the TDM strategy in clinical settings and also from the perspective of pharmacovigilance. Indeed, multiple publications in this field have concluded that the same assay should be used for longitudinal follow-up of a patient, as the kits are not interchangeable notably because of the lack of standardization (59, 60). However, a study limitation is that all serum samples tested contained none or undetectable levels of infliximab which may not adequately reflect the clinical scenario.

ADAs induced initially in patients following treatment with a biotherapeutic are typically low in titer, affinity, and avidity and mature over time into stronger-binding high titer immunoglobulins as progressive therapy continues. Consequently, both low- and high-affinity ADAs must be detectable in ADA assays used in clinical practice. It is acknowledged that the use of surrogate monoclonal antibodies as representative of a polyclonal response that is induced in patients is not ideal and their use as calibrators is contrary to regulatory practice (27, 28). However, there is a need as explained earlier for ADA standard(s) for use in clinical monitoring assays and therefore based on the study data as well as their reactivity with biosimilars (e.g., Remsima, Flixabi) tested, it was considered important that B, an antibody with a fast dissociation rate is also provided to users (for assessing assay performance) along with A which is a high affinity antibody (for use in assay performance, validation, calibration).

The data clearly illustrated the suitability of 19/234 (A) and 19/232 (B) as the WHO International Reference Panel for Infliximab anti-drug antibodies and indicated that reporting of results using a common antibody reference standard is likely to align results (possibly even allow classification of low/medium and high titers to be consistently established) and allow comparisons from use of different assays. Moreover, it would also enable harmonization of assays compared with the existing situation where reporting units are not comparable (even if stated as ng/ml or µg/ml). Given the limited experience to date with use of the panel, the use of the same assay longitudinally when following patient ADA response would still be the preferred option. Only 27% of the participating laboratories reported in arbitrary units or titres in this study but there are an increasing number of commercial kits in use for clinical monitoring with disparate reporting. This data was presented to the WHO Expert Committee on Biological standardization (ECBS) at its meeting in October 2022. The committee recommended the following: (1) the establishment of the WHO International Reference Panel containing two samples A coded 19/234 and B coded 19/232 for distribution and use by the scientific community. (2) Sample A (19/234) to serve as a ‘common standard’ for assay characterization and for calibration of in-house preparations and commercially available anti-infliximab ADAs with an arbitrarily assigned unitage of 50,000 IU/ampoule for binding activity and 50,000 IU/ampoule for neutralising activity. This would facilitate comparison of results across immunogenicity assays, if implemented in practice and aid TDM for better patient outcome globally. Sample B (19/232) based on its unique characteristics to serve an important role in assessing whether the envisaged assay is capable of detecting ADAs with fast dissociation. However, no unitage was assigned to reference preparation B. This panel is available from MHRA, UK which serves as the custodian laboratory for a wide range of WHO standards.

## 5 Conclusions

A panel of two human mAbs against infliximab with defined characteristics - varied isotypes, different binding characteristics but both with neutralizing activity was formulated, lyophilized and assessed in an international study. The study indicated the utility and suitability of the panel containing mAbs A coded 19/234 and B coded 19/232 as a WHO international reference panel for infliximab ADA assays. The use of this panel by industry would support TDM and likely impact treatment regimens with improvement in patient disease outcome globally.

## Data Availability

The original contributions presented in the study are included in the article/**Supplementary Material**. Further inquiries can be directed to the corresponding author.

## Group members of Study Participants

Zehra Arkir, Jenny Leung and Krystal Rawstron, Viapath Analytics, Reference Chemistry Laboratory, St Thomas’ Hospital, 4th floor, North Wing, London SE1 7EH, UK; Tom Lourens, Karien Bloem, Floris Loeff, Sanquin Diagnostic Services, Biologicals Laboratory, Plesmanlaan 125, 1066CX, Amsterdam, Netherlands; Begoña Ruiz-Argüello and Daniel Nagore, Progenika Biopharma S.A., Parque Tecnológico Bizkaia, Ed. 504, 48160 Derio-Bizkaia, Spain; Guillaume Noguier, Ermis Parussini and Simon Davière, Theradiag, 14 Rue Ambroise Croizat, CS90136 Croissy Beaubourg, 77435 Marne la Vallee, France; Michael Schneider and Thomas Schuster, BÜHLMANN Laboratories AG, Baselstrasse 55, 4124 Schönenbuch, Switzerland; Isabelle Cludts and Meenu Wadhwa, NIBSC-MHRA, Blanche Lane, Potters Bar, Hertfordshire EN6 3QG, UK; Melissa Snyder, Cory Blixt and Mary Pido, Mayo Clinic, Department of Laboratory Medicine and Pathology 200 First Street SW, Ro-Hi-2-10-PI, Rochester, MN 55905, USA; Aparna Kasinath and Raghu Vandavagali, Syngene International Ltd, Tower 1, Semicon Park, Hosur Road, Electronic City – Phase 2, Bangalore 560004, India; Peter Galloway and Allan Dunlop, Queen Elizabeth University Hospital, Biochemistry lab, Glasgow G51 4TF, UK; Alexandra Thurston-Postle, Rachel Griffiths and Adedoja Adeseye, Manuals Laboratory, Pathology, Sandwell General Hospital, Lyndon, West Bromwich B71 4HJ, UK; Takuo Suzuki, Minoru Tada and Akiko Ishii-Watabe, National Institute of Health Sciences, Division of Biological Chemistry and Biologicals, 3-25-26 Tonomachi, Kawasaki-ku, Kawasaki, Kanagawa, 210-9501, Japan; Paul Declerck and Debby Thomas, KUL, Laboratory Therapeutic and Diagnostic Antibodies, Campus GHB, Herestraat 49, 3000 Leuven, Belgium; Lone Frier Bovin and Karin Blume, Svar Life Science, Lundavägen 151, SE-21224 Malmö, Sweden; Anna Fogdell-Hahn, Anna Mattsson, Nastya Kharlamova, Karolinska Institutet, Department of Clinical Neuroscience, Center for Molecular Medicine (CMM), Visionsgatan 18, Solna, SE-171 76 Stockholm, Sweden; Natacha Szely and Marc Pallardy, Unité INSERM UMR 996, rue J.B. Clément, Châtenay-Malabry 92296, France; Katrin Deiser, Lisa Allmannsberger and Gregor Schaffar, Sandoz Clinical Bioanalytics, Hexal AG, Keltenring 1 + 3, Oberhaching, 82041, Germany; Erin Summers Bellin, Catherine Vrentas, Katelynn Brann, Madeleine Palumbo and Elyse Messick, PPD Laboratories, 2251 Dabney Road, Suite I, Richmond, VA 23230, USA
