# Supplementary material for: The first WHO reference panel for Infliximab anti-drug antibodies: a step towards harmonizing therapeutic drug monitoring
Source: Front Immunol. 2025 Mar 20;16:1550655. doi: 10.3389/fimmu.2025.1550655 (PMC11965635; doi:10.3389/fimmu.2025.1550655)
Supplement: Supplementary file 1 [file DataSheet1.docx]

**Supplemental Data:**

**The first WHO reference panel for Infliximab anti-drug antibodies: A step towards harmonizing therapeutic drug monitoring**

**Meenu Wadhwa^1*^, Isabelle Cludts^1^, Eleanor Atkinson^2^ and Peter Rigsby^2^ on behalf of Study Participants**

^1^ Biotherapeutics and Advanced therapies Group, R&D Division, Science and Research, Medicines and HealthCare products Regulatory agency (MHRA), Blanche Lane, South Mimms, Potters Bar, HERTS, EN6 3QG

^2^ Analytical and Biological Sciences Group, R&D Division, Science and Research, Medicines and HealthCare products Regulatory agency (MHRA), Blanche Lane, South Mimms, Potters Bar, HERTS, EN6 3QG

**ABIRISK mAbs:** **Methods for isolation and characterization of anti-drug antibodies against monoclonal antibodies (IRB, Switzerland)**

**Biopharmaceutical products (BPs) used as antigens for different assays.** Rebif (Merck Serono) was used as source of IFN-beta. Rituximab, Natalizumab, Adalimumab and Infliximab were produced recombinantly as chimeric human-IgG1(CH1)-mouse-IgG2a(CH2-CH3) to avoid cross-reaction of secondary anti-human Fc-specific antibodies used to detect binding of human monoclonal antibodies.

**Isolation and production of monoclonal antibodies from patients with serum ADAs.** Peripheral blood samples were obtained from treated patient. Memory B cells were isolated from cryopreserved PBMCs using anti-FITC microbeads (Miltenyi Biotec) following staining of PBMCs with CD22-FITC (BD Phamingen), and were immortalized with Epstein-Barr virus and CpG in multiple wells as described previously [^1^](file:///C:\Users\mwadhwa\AppData\Local\Microsoft\Windows\INetCache\Content.Outlook\BKPS7YUE\Methods%20IRB%20(002).docx#_ENREF_1). Culture supernatants were tested for binding to specific BPs by ELISA. cDNA was synthesized from positive cultures and both heavy chain and light chain variable regions were sequenced. Positive cultures were expanded and supernatants were collected and purified. When positive cultures could not be expanded, monoclonal antibodies were produced recombinantly as IgG1 by transient transfection of Expi293 cells (Invitrogen) using polyethylenimine (PEI) and tested for binding to Infliximab.

**Sequence analysis of antibodies.**

The usage of VH and VL genes and the amount of somatic mutations were determined by analyzing the homology of VH and VL sequences of mAbs to known human V, D and J genes by the IMGT (international ImMunoGeneTics information system) database [^2^](file:///C:\Users\mwadhwa\AppData\Local\Microsoft\Windows\INetCache\Content.Outlook\BKPS7YUE\Methods%20IRB%20(002).docx#_ENREF_2).

**Antibody purification.** Chimeric BPs and human mAbs were purified by protein A or protein G chromatography (GE Healthcare) and concentrated by Amicon Ultra filter units (100K, Millipore). Total IgGs were quantified by Pierce BCA protein assay (Thermofischer).

**Scaled up Antibody production.** To enable scaled up production, antibodies were cloned in a stable recombinant Chinese hamster ovary (rCHO) cell line.  The antibodies were expressed from these suspension-cultured rCHO cell lines (not clonal cell line). Culture supernatants containing the secreted protein were harvested ten to fourteen days post-inoculum, centrifuged and filtered on a 0.22 µm membrane. Antibody was purified by affinity chromatography on Protein A (MabSelectsure, GE Healthcare) using acidic conditions for elution. Subsequently, the pool of fractions containing the antibody was purified by size exclusion chromatography (Superdex 200, GE Healthcare) equilibrated with DPBS. The first set of purification was done on an automated platform at 10 mg “small-scale”. All the characterization data obtained for this “small-scale” production was reviewed, along with expression yields, and a selection of antibodies was made for “large-scale” production. Ten clones were produced at 0.5-1 g scale-up. Production was similar in process to the “small-scale” however the purification, was performed on larger chromatography columns using a parallel device system. Purified antibodies were transferred to the NIBSC. rCHO cell lines are kept with Sanofi.

**ELISA assays.**

Binding to BPs was tested by ELISA using 384-well SpectraPlates (PerkinElmer) for primary screenings or 96-well MaxiSorp plates (Nunc) for any following test. Briefly, ELISA plates were coated with 1 μg/ml of BP, blocked with 1% BSA and incubated with titrated antibodies, followed by AP-conjugated anti-human IgG - Fc gamma specific secondary antibodies (Jackson ImmunoResearch). Plates were then washed, substrate (p-NPP, Sigma) was added and plates were read at 405 nm. EC50 (ng/ml) was calculated for every sample by nonlinear regression analysis using GraphPad Prism 5 software.

**Surface plasmon resonance (SPR) assays.** Monoclonal antibodies (50 nM) were stabilized in 10 mM acetate buffer, pH 4.5, and immobilized onto a EDC/NHS pre-activated ProteOn sensor chip (Biorad) through amine coupling; unreacted groups were blocked by injection of ethanolamine HCl (1 M). HEPES buffered saline (HBS) (10 mM HEPES, pH 7.4, 150 mM NaCl, 3 mM EDTA, 0.005% surfactant Tween-20) was used as running buffer. All injections were made at flow rate of 100 μl/min. M were diluted and titrated in HBS (90-30-10-3.3-1.1 nM) and injected onto the BP coated chip; one channel of the chip was injected with HBS and used as reference for the analysis. Injection time and dissociation time were 240 s and 900 s, respectively. Each binding interaction of mAbs was assessed using a ProteON XPR36 instrument (Biorad) and data processed with ProteOn Manager Software. Ka, Kd and KD were calculated applying the Langmuir fit model.

**Neutralization assays.**

ELISA plates were coated with 2 μg/ml of TNF-alpha (antibody target) and blocked with 1% BSA. Chimeric Infliximab was diluted to 60 ng/ml (final dilution) and incubated with titrated monoclonal antibodies for 1 h, 37°C. The mixes were transferred to the ELISA plates and incubated for 90 min, RT, followed by AP-conjugated anti-mouse IgG (Southern Biotech). Plates were then washed, substrate (p-NPP, Sigma) was added and plates were read at 405 nm.

Neutralisation was calculated as percentage of inhibition to TNF-alpha with the following formula: [1-(OD of a single well – average OD of control cells incubated without Infliximab)/(average OD of control cells incubated with Infliximab – average OD of control cells incubated without Infliximab)] x 100. IC90 (ng/ml) was calculated for every sample by a nonlinear regression analysis using GraphPad Prism 5 software.

**References.**

1. Traggiai, E.*, et al.* An efficient method to make human monoclonal antibodies from memory B cells: potent neutralisation of SARS coronavirus. *Nature medicine* **10**, 871-875 (2004).

2. Lefranc, M.P.*, et al.* IMGT, the international ImMunoGeneTics information system. *Nucleic acids research* **37**, D1006-1012 (2009).

**Table 1:** **Details of clinical serum samples**

| **Sample code** | **ADA level (anticipated)** | **ADA level*** |
| --- | --- | --- |
| **1** | undetectable | Pooled sera with ADA titers <10AU/ml (equal ratio) |
| **2** | low - mod | Pooled sera with ADA titers of 21-100 and 101-200AU/ml (1:4 ratio) |
| **3** | moderate | Pooled sera with ADA titers of 21-60, 201-400 AU/ml (equal ratio) |
| **4** | high | Pooled sera with ADA titers >400 AU/ml (equal ratio) |
| **5** | low - mod | Pooled sera with ADA titer >400 AU/ml, diluted 1:40 in nhs |
| **6** | low - mod | pool of serum samples with ADA titer >400 AU/ml, diluted 1:30 in nhs |
| **7** | negative | normal human serum (First Link, sterile mixed pool, #20-00-850, batch HSS8963 |

*ADA titers determined by ELISA in the hospital providing the samples; nhs – normal human serum

**Table 2: Assay data for mAb preparations (top) and serum samples (bottom)**

| **ECL - Calculated vs A (μg/ml)** | | | | | |  |  | | |  | | |  | | |  | | |  | |  |  | |  | |  |  |  |
| --- | --- | --- | --- | --- | --- | --- | --- | --- | --- | --- | --- | --- | --- | --- | --- | --- | --- | --- | --- | --- | --- | --- | --- | --- | --- | --- | --- | --- |
|  | | | |  | | |  | | |  | | |  | | |  | | |  | |  |  | |  | |  |  |  |
| **Sample** | **Lab** | | | | | | | | | | | | | | | **Range** | | | **GM** | | **GCV** | **GM***** | | **GCV***** | |  |  |  |
|  | **9** | | **10** | | **11** | | | **12** | | | **13** | | | **17** | |  |  |  |  |  |  |  |  |  |  |  |  |  |
| **mAbs** |  | |  | |  | | |  | | |  | | |  | |  | | |  | |  |  | |  | |  |  |  |
| **A** | 50.00 | | 50.00 | | 50.00 | | | 50.00 | | | 50.00 | | | 50.00 | |  | | |  | |  |  | |  | |  |  |  |
| **P** | 38.32 | | 11.73 | | 36.98 | | | 44.66 | | | 26.91 | | | 8.47 | | 8.47 - 44.66 | | | 23.52 | | 100% | 28.85 | | 71% | |  |  |  |
| **O** | 37.62 | | 8.06 | | 34.58 | | | 57.74 | | | 22.05 | | | 11.85 | | 8.06 - 57.74 | | | 23.26 | | 111% | 26.62 | | 112% | |  |  |  |
| **S** | 29.31 | | 39.56 | | 45.37 | | | 47.04 | | | 33.86 | | | 36.61 | | 29.31 - 47.04 | | | 38.12 | | 20% | 38.43 | | 22% | |  |  |  |
| **B** | 25.31 | | n/t | | 38.35 | | | 46.24 | | | 95.42 | | | 105.87 | | 25.31 - 105.87 | | | 53.86 | | 84% | 45.49 | | 74% | |  |  |  |
| **N** | 6.87 | | n/t | | 8.54 | | | 9.37 | | | 12.79 | | | 14.84 | | 6.87 - 14.84 | | | 10.09 | | 36% | 9.16 | | 29% | |  |  |  |
| **R** | 1.89 | | NP | | 10.33 | | | 7.04 | | | 7.19 | | | 0.63 | | 0.63 - 10.33 | | | 3.62 | | 222% | 5.61 | | 111% | |  |  |  |
| **Q** | 2.60 | | 4.49 | | 2.62 | | | NP | | | NP | | | 0.49 | | 0.49 - 4.49 | | | 1.97 | | 162% | 3.13 | | 37% | |  |  |  |
| **sera** |  | |  | |  | | |  | | |  | | |  | |  | | |  | |  |  | |  | |  |  |  |
| **4** | 12.49 | | n/t | | n/t | | | 5.02 | | | 8.39 | | | 22.57 | | 5.02 - 22.57 | | | 10.44 | | 89% | 8.07 | | 58% | |  |  |  |
| **3** | 10.25 | | n/t | | n/t | | | 1.95 | | | 8.64 | | | 19.17 | | 1.95 - 19.17 | | | 7.59 | | 163% | 5.57 | | 149% | |  |  |  |
| **6** | 0.51 | | n/t | | n/t | | | 3.89 | | | 0.35 | | | 0.61 | | 0.35 - 3.89 | | | 0.81 | | 193% | 0.88 | | 267% | |  |  |  |
| **5** | 0.39 | | n/t | | n/t | | | 2.01 | | | 0.26 | | | 0.51 | | 0.26 - 2.01 | | | 0.56 | | 144% | 0.58 | | 198% | |  |  |  |
| **2** | 0.30 | | n/t | | n/t | | | 0.16 | | | 0.11 | | | 0.41 | | 0.11 - 0.41 | | | 0.22 | | 79% | 0.18 | | 64% | |  |  |  |
| **1** | Neg | | Neg | | Neg | | | Neg | | | Neg | | | Neg | |  | | |  | |  |  | |  | |  |  |  |
| **7** | Neg | | Neg | | Neg | | | Neg | | | Neg | | | Neg | |  | | |  | |  |  | |  | |  |  |  |
|  |  |  | |  | | |  | | |  | | |  | | |  | | |  | |  |  | |  | |  |  |  |
| **ECL - Calculated vs IH/kit standards** | | | | | | | | |  | | |  | | |  | |  |  | |  | | |  | |  | |  |  |
| **Sample** | **Lab** | | | | | | | | | | | | | | | **Range** | | | **GM** | | **GCV** | **GM***** | | **GCV***** | |  |  |  |
|  | **9** | **10** | | **11** | | | **12** | | | **13** | | | **17** | | |  |  |  |  |  |  |  |  |  |  |  |  |  |
| **mAbs** |  |  | |  | | |  | | |  | | |  | | |  | | |  | |  |  | |  | |  |  |  |
| **A** | 6.55 | 15.68 | | 246.05 | | | 30.89 | | | 12.12 | | | 113.40 | | | 6.55 - 246.05 | | | 32.00 | | 303% | 24.84 | | 304% | |  |  |  |
| **P** | 5.02 | 3.68 | | 183.97 | | | 31.72 | | | 5.75 | | | 17.78 | | | 3.68 - 183.97 | | | 14.92 | | 340% | 14.41 | | 422% | |  |  |  |
| **O** | 4.93 | 2.53 | | 172.04 | | | 37.31 | | | 4.72 | | | 27.63 | | | 2.53 - 172.04 | | | 14.78 | | 398% | 13.04 | | 483% | |  |  |  |
| **S** | 3.87 | 12.41 | | 220.85 | | | 24.97 | | | 7.24 | | | 76.79 | | | 3.87 - 220.85 | | | 22.98 | | 355% | 18.05 | | 375% | |  |  |  |
| **B** | 3.32 | 3.44 | | 190.80 | | | 27.90 | | | 24.81 | | | 246.84 | | | 3.32 - 246.84 | | | 26.81 | | 545% | 17.19 | | 444% | |  |  |  |
| **N** | 0.89 | n/t | | 42.49 | | | 5.66 | | | 3.33 | | | 34.60 | | | 0.89 - 42.49 | | | 7.56 | | 410% | 9.19 | | 200% | |  |  |  |
| **R** | 0.25 | 1.07 | | 50.28 | | | 4.46 | | | 1.54 | | | 1.51 | | | 0.25 - 50.28 | | | 2.27 | | 493% | 3.91 | | 359% | |  |  |  |
| **Q** | 0.34 | 1.47 | | 12.77 | | | 2.61 | | | 0.55 | | | 1.17 | | | 0.34 - 12.77 | | | 1.49 | | 258% | 2.47 | | 217% | |  |  |  |
| **sera** |  |  | |  | | |  | | |  | | |  | | |  | | |  | |  |  | |  | |  |  |  |
| **4** | 1.63 | 4.02 | | n/t | | | 3.18 | | | 2.33 | | | 53.83 | | | 1.63 - 53.83 | | | 4.83 | | 301% | 2.64 | | 48% | |  |  |  |
| **3** | 1.34 | 4.37 | | n/t | | | 1.24 | | | 2.47 | | | 45.72 | | | 1.24 - 45.72 | | | 3.82 | | 338% | 2.06 | | 80% | |  |  |  |
| **6** | 0.07 | 0.06 | | n/t | | | 2.46 | | | 0.09 | | | 1.29 | | | 0.06 - 2.46 | | | 0.26 | | 497% | 0.17 | | 495% | |  |  |  |
| **5** | 0.05 | 0.05 | | n/t | | | 1.27 | | | 0.06 | | | 1.06 | | | 0.05 - 1.27 | | | 0.19 | | 428% | 0.12 | | 378% | |  |  |  |
| **2** | 0.05 | 0.25 | | n/t | | | 0.10 | | | 0.03 | | | 0.95 | | | 0.03 - 0.95 | | | 0.13 | | 300% | 0.08 | | 155% | |  |  |  |
| **1** | Neg | Neg | | Neg | | | Neg | | | Neg | | | Neg | | |  | | |  | |  |  | |  | |  |  |  |
| **7** | Neg | Neg | | Neg | | | Neg | | | Neg | | | Neg | | |  | | |  | |  |  | |  | |  |  |  |
| **Units** | μg/ml | μg/ml | | μg/ml | | | μg/ml | | | μg/ml | | | μg/ml | | |  | | |  | |  |  | |  | |  |  |  |

| **Other assays – Calculated vs A (μg/ml)** | | | | | | | | | | | |  | |  | |  | |  |  |
| --- | --- | --- | --- | --- | --- | --- | --- | --- | --- | --- | --- | --- | --- | --- | --- | --- | --- | --- | --- |
|  | | | | |  | | |  | | |  | | | |  |  |  |  |  |
| **Sample** | **Lab** | | | | | | | | | | **Range LF (1b&15)** | | | |  |  |  |  |  |
|  | **1b** | **15** | | **2b** | | | | | **14** | |  |  |  |  |  |  |  |  |  |
| **mAbs** |  |  | |  | | | | |  | |  | | | |  |  |  |  |  |
| **A** | 50.00 | 50.00 | | 50.00 | | | | | 50.00 | |  | | | |  |  |  |  |  |
| **P** | 12.60 | 14.70 | | 12.98 | | | | | 14.96 | | 12.60-14.70 | | | |  |  |  |  |  |
| **O** | 7.94 | 12.40 | | 10.35 | | | | | 17.60 | | 7.94 - 12.40 | | | |  |  |  |  |  |
| **S** | 12.60 | 9.57 | | 13.60 | | | | | 20.02 | | 9.57 - 12.60 | | | |  |  |  |  |  |
| **B** | 15.75 | 43.71 | | 11.87 | | | | | 8.04 | | 15.75 - 43.71 | | | |  |  |  |  |  |
| **N** | 7.94 | 8.52 | | 6.03 | | | | | 10.13 | | 7.94 -8.52 | | | |  |  |  |  |  |
| **R** | Pos | 0.4 | | 0.04 | | | | | 1.44 | |  | | | |  |  |  |  |  |
| **Q** | Pos | 0.2 | | 0.02 | | | | | 1.27 | |  | | | |  |  |  |  |  |
| **sera** |  |  | |  | | | | |  | |  | | | |  |  |  |  |  |
| **4** | 6.20 | 8.97 | | 3.37 | | | | | 19.19 | | 6.20 - 8.97 | | | |  |  |  |  |  |
| **3** | 6.20 | 11.32 | | 2.78 | | | | | 27.34 | | 6.20 - 11.32 | | | |  |  |  |  |  |
| **6** | 0.16 | 0.2 | | 0.12 | | | | | 0.44 | | 0.16-0.20 | | | |  |  |  |  |  |
| **5** | 0.08 | 0.2 | | 0.08 | | | | | 0.32 | | 0.08-0.20 | | | |  |  |  |  |  |
| **2** | 0.08 | 0.2 | | 0.03 | | | | | 0.61 | | 0.08-0.20 | | | |  |  |  |  |  |
| **1** | Neg | Neg | | Neg | | | | | Neg | |  | | | |  |  |  |  |  |
| **7** | Neg | Neg | | Neg | | | | | Neg | |  | | | |  |  |  |  |  |
|  |  | |  | |  | | |  | | |  | | | |  |  |  |  |  |
| **Other assays – Calculated vs IH** | | | | | | | | | | | |  | | | |  |  | |  |
| **Sample** | **Lab** | | | | | | | | | |  | | | |  |  |  |  |  |
|  | **1b** | **15** | | **2b** | | | | | **14** | |  | | | |  |  |  |  |  |
| **mAbs** |  |  | |  | | | | |  | |  | | | |  |  |  |  |  |
| **A** | 4031.75 | 52.90 | | 594.14 | | | | | 9100.13 | |  | | | |  |  |  |  |  |
| **P** | 1015.94 | 15.57 | | 151.92 | | | | | 2722.50 | |  | | | |  |  |  |  |  |
| **O** | 640.00 | 15.22 | | 121.79 | | | | | 3203.49 | |  | | | |  |  |  |  |  |
| **S** | 1015.94 | 10.10 | | 159.11 | | | | | 3643.72 | |  | | | |  |  |  |  |  |
| **B** | 1269.92 | 46.25 | | 139.89 | | | | | 1463.53 | |  | | | |  |  |  |  |  |
| **N** | 640.00 | 9.01 | | 71.46 | | | | | 1843.75 | |  | | | |  |  |  |  |  |
| **R** | >=8 | 0.45 | | 0.50 | | | | | 262.22 | |  | | | |  |  |  |  |  |
| **Q** | 5.04 | 0.21 | | 0.25 | | | | | 230.43 | |  | | | |  |  |  |  |  |
| **sera** |  |  | |  | | | | |  | |  | | | |  |  |  |  |  |
| **4** | 500.00 | 9.48 | | 38.27 | | | | | 3452.43 | |  | | | |  |  |  |  |  |
| **3** | 500.00 | 11.96 | | 26.37 | | | | | 5190.43 | |  | | | |  |  |  |  |  |
| **6** | 12.70 | 0.21 | | 1.40 | | | | | 79.41 | |  | | | |  |  |  |  |  |
| **5** | 6.35 | 0.27 | | 0.94 | | | | | 57.58 | |  | | | |  |  |  |  |  |
| **2** | 6.35 | 0.21 | | 0.34 | | | | | 111.78 | |  | | | |  |  |  |  |  |
| **1** | Neg | Neg | | Neg | | | | | Neg | |  | | | |  |  |  |  |  |
| **7** | Neg | Neg | | Neg | | | | | Neg | |  | | | |  |  |  |  |  |
| Units | titre | | μg/ml | | μg/ml | | | AU/ml | | |  | | | |  |  |  |  |  |
|  | | | | | |  |  |  | |  | | |  |  |  |  |  |  |  |

| **Neutralisation assays – Calculated vs A (μg/ml)** | | | | | | | | | | | | | | | | | | | | | | | | | |  |  | | |  | | |  | |  | | |  | | |  | |
| --- | --- | --- | --- | --- | --- | --- | --- | --- | --- | --- | --- | --- | --- | --- | --- | --- | --- | --- | --- | --- | --- | --- | --- | --- | --- | --- | --- | --- | --- | --- | --- | --- | --- | --- | --- | --- | --- | --- | --- | --- | --- | --- |
|  | | | | | |  | |  | |  | |  | | | |  | | |  | | | |  | | | |  |  |  |  |  |  |  |  |  |  |  |  |  |  |  |  |
| **Sample** | **Lab** | | | | | | | | | **Range** | | **GM** | | | | | **GCV** | | **GM***** | | | **GCV***** | | | | |  |  |  |  |  |  |  |  |  |  |  |  |  |  |  |  |
|  | **8c** | | **8b** | | **16** | | **17** | | |  |  |  |  |  |  |  |  |  |  |  |  |  |  |  |  |  |  |  |  |  |  |  |  |  |  |  |  |  |  |  |  |  |
| **mAbs** |  | |  | |  | |  | | |  | |  | | | | |  | |  | | |  | | | | |  |  |  |  |  |  |  |  |  |  |  |  |  |  |  |  |
| **A** | 50.00 | | 50.00 | | 50.00 | | 50.00 | | |  | |  | | | | |  | |  | | |  | | | | |  |  |  |  |  |  |  |  |  |  |  |  |  |  |  |  |
| **P** | 8.98 | | 13.30 | | 9.94 | | 2.85 | | | 2.85-13.30 | | 7.63 | | | | | 97% | | 10.59 | | | 23% | | | | |  |  |  |  |  |  |  |  |  |  |  |  |  |  |  |  |
| **O** | 9.63 | | 13.85 | | 9.18 | | 7.49 | | | 7.49-13.85 | | 9.79 | | | | | 29% | | 10.70 | | | 25% | | | | |  |  |  |  |  |  |  |  |  |  |  |  |  |  |  |  |
| **S** | 11.40 | | 23.60 | | 9.18 | | 13.20 | | | 9.18-23.60 | | 13.44 | | | | | 50% | | 13.52 | | | 64% | | | | |  |  |  |  |  |  |  |  |  |  |  |  |  |  |  |  |
| **B** | 6.94 | | 11.11 | | 36.96 | | NP | | | 6.94-36.96 | | 14.18 | | | | | 137% | | 14.18 | | | 137% | | | | |  |  |  |  |  |  |  |  |  |  |  |  |  |  |  |  |
| **N** | 4.97 | | 6.01 | | 6.97 | | NP | | | 4.97-6.97 | | 5.93 | | | | | 18% | | 5.93 | | | 18% | | | | |  |  |  |  |  |  |  |  |  |  |  |  |  |  |  |  |
| **R** | 1.69 | | NP | | NP | | NP | | |  | | 1.69 | | | | |  | | 1.69 | | |  | | | | |  |  |  |  |  |  |  |  |  |  |  |  |  |  |  |  |
| **Q** | 1.48 | | NP | | NP | | NP | | |  | | 1.48 | | | | |  | | 1.48 | | |  | | | | |  |  |  |  |  |  |  |  |  |  |  |  |  |  |  |  |
| **sera** |  | |  | |  | |  | | |  | |  | | | | |  | |  | | |  | | | | |  |  |  |  |  |  |  |  |  |  |  |  |  |  |  |  |
| **4** | 9.65 | | 14.00 | | 12.13 | | NP | | | 9.65-14.00 | | 11.79 | | | | | 21% | | 11.79 | | | 21% | | | | |  |  |  |  |  |  |  |  |  |  |  |  |  |  |  |  |
| **3** | 15.27 | | 19.40 | | 21.77 | | NP | | | 15.27-21.77 | | 18.61 | | | | | 20% | | 18.61 | | | 20% | | | | |  |  |  |  |  |  |  |  |  |  |  |  |  |  |  |  |
| **6** | 0.24 | | 0.22 | | 0.31 | | 0.29 | | | 0.22-0.31 | | 0.26 | | | | | 16% | | 0.26 | | | 18% | | | | |  |  |  |  |  |  |  |  |  |  |  |  |  |  |  |  |
| **5** | 0.17 | | 0.14 | | NP | | 0.22 | | | 0.14-0.22 | | 0.17 | | | | | 27% | | 0.15 | | | 15% | | | | |  |  |  |  |  |  |  |  |  |  |  |  |  |  |  |  |
| **2** | 0.44 | | 0.40 | | NP | | NP | | | 0.40-0.44 | | 0.42 | | | | |  | | 0.42 | | |  | | | | |  |  |  |  |  |  |  |  |  |  |  |  |  |  |  |  |
| **1** | Neg | | Neg | | Neg | | Neg | | |  | |  | | | | |  | |  | | |  | | | | |  |  |  |  |  |  |  |  |  |  |  |  |  |  |  |  |
| **7** | Neg | | Neg | | Neg | | Neg | | |  | |  | | | | |  | |  | | |  | | | | |  |  |  |  |  |  |  |  |  |  |  |  |  |  |  |  |
|  |  | | |  | |  | |  | |  | |  | | | |  | | |  | | | |  | | | |  |  |  |  |  |  |  |  |  |  |  |  |  |  |  |  |
| **Neutralisation assays - calculated vs IH/kit standards** | | | | | | | | | | | | | |  | | | |  | | |  | | |  | | | |  | | |  | | |  | | |  | |  |  |  |  |
| **Sample** | **Lab** | | | | | | | | | **range**** | | **GM**** | | | | | **GCV**** | | **GM***** | | | **GCV***** | | | | |  |  |  |  |  |  |  |  |  |  |  |  |  |  |  |  |
|  | **8c** | | **8b** | | **16^1^** | | **17** | | |  |  |  |  |  |  |  |  |  |  |  |  |  |  |  |  |  |  |  |  |  |  |  |  |  |  |  |  |  |  |  |  |  |
| **mAbs** |  | |  | |  | |  | | |  | |  | | | | |  | |  | | |  | | | | |  |  |  |  |  |  |  |  |  |  |  |  |  |  |  |  |
| **A** | 57.67 | | 24.31 | | 9.10 | | 40.90 | | | 24.31-57.67 | | 38.56 | | | | | 54% | | 37.44 | | | 84% | | | | |  |  |  |  |  |  |  |  |  |  |  |  |  |  |  |  |
| **P** | 10.26 | | 6.44 | | 12.75 | | 2.00 | | | 2.00-10.26 | | 5.09 | | | | | 132% | | 8.13 | | | 39% | | | | |  |  |  |  |  |  |  |  |  |  |  |  |  |  |  |  |
| **O** | 11.00 | | 6.13 | | 16.00 | | 5.95 | | | 5.95-11.00 | | 7.37 | | | | | 41% | | 8.21 | | | 51% | | | | |  |  |  |  |  |  |  |  |  |  |  |  |  |  |  |  |
| **S** | 12.68 | | 8.02 | | 12.75 | | 9.25 | | | 8.02-12.68 | | 9.80 | | | | | 26% | | 10.08 | | | 38% | | | | |  |  |  |  |  |  |  |  |  |  |  |  |  |  |  |  |
| **B** | 8.27 | | 6.09 | | n/t | | NP | | | 6.09-8.27 | | 7.10 | | | | |  | | 7.1 | | |  | | | | |  |  |  |  |  |  |  |  |  |  |  |  |  |  |  |  |
| **N** | 5.92 | | 3.38 | | 16.00 | | NP | | | 3.38-5.92 | | 4.47 | | | | |  | | 4.47 | | |  | | | | |  |  |  |  |  |  |  |  |  |  |  |  |  |  |  |  |
| **R** | 1.88 | | 0.17 | | 33.00 | | NP | | | 0.17-1.88 | | 0.56 | | | | |  | | 0.56 | | |  | | | | |  |  |  |  |  |  |  |  |  |  |  |  |  |  |  |  |
| **Q** | 1.90 | | 0.24 | | 26.00 | | NP | | | 0.24-1.90 | | 0.67 | | | | |  | | 0.67 | | |  | | | | |  |  |  |  |  |  |  |  |  |  |  |  |  |  |  |  |
| **sera** |  | |  | |  | |  | | |  | |  | | | | |  | |  | | |  | | | | |  |  |  |  |  |  |  |  |  |  |  |  |  |  |  |  |
| **4** | 11.30 | | 6.02 | | 1016 | | NP | | | 6.02-11.30 | | 8.05 | | | | | 56% | |  | | |  | | | | |  |  |  |  |  |  |  |  |  |  |  |  |  |  |  |  |
| **3** | 17.89 | | 8.63 | | 2031 | | NP | | | 8.63-17.89 | | 12.43 | | | | | 67% | |  | | |  | | | | |  |  |  |  |  |  |  |  |  |  |  |  |  |  |  |  |
| **6** | 0.28 | | 0.10 | | 40 | | NP | | | 0.10-0.28 | | 0.17 | | | | | 109% | |  | | |  | | | | |  |  |  |  |  |  |  |  |  |  |  |  |  |  |  |  |
| **5** | 0.20 | | 0.09 | | 20 | | NP | | | 0.09-0.20 | | 0.13 | | | | | 71% | |  | | |  | | | | |  |  |  |  |  |  |  |  |  |  |  |  |  |  |  |  |
| **2** | 0.52 | | 0.17 | | 80 | | NP | | | 0.17-0.52 | | 0.3 | | | | | 120% | |  | | |  | | | | |  |  |  |  |  |  |  |  |  |  |  |  |  |  |  |  |
| **1** | Neg | | Neg | | Neg | | Neg | | |  | |  | | | | |  | |  | | |  | | | | |  |  |  |  |  |  |  |  |  |  |  |  |  |  |  |  |
| **7** | Neg | | Neg | | Neg | | Neg | | |  | |  | | | | |  | |  | | |  | | | | |  |  |  |  |  |  |  |  |  |  |  |  |  |  |  |  |
| Units | μg/ml | | | μg/ml | | titer | | μg/ml | |  | |  | | | |  | | |  | | | |  | | | |  |  |  |  |  |  |  |  |  |  |  |  |  |  |  |  |
|  | |  | | | | | | |  | |  | |  | |  | | | | |  | | | | |  | | | |  | | |  | | | |  | | | |  | |  |
| NP | | Non-parallel to standard | | | | | | | | | | |  | |  | | | | |  | | | | |  | | | |  | | |  | | | |  | | | |  | |  |
| n/t | | Sample not tested on same plates as standard | | | | | | | | | | | | | | | | | |  | | | | |  | | | |  | | |  | | | |  | | | |  | |  |
| n/t | | Sample not tested by lab | | | | | | | | | | |  | |  | | | | |  | | | | |  | | | |  | | |  | | | |  | | | |  | |  |
| x | | Sample reported as negative or below assay lower quantitation limit | | | | | | | | | | | | | | | | | | | | | | | | | | |  | | |  | | | |  | | | |  | |  |
| x | | Sample reported as positive but no quantitative estimate available (e.g., above assay upper quantitation at dilution(s) tested) | | | | | | | | | | | | | | | | | | | | | | | | | | | | | | | | | | | | | | | |  |
| * | | GM and GCV also calculated with exclusion of these labs | | | | | | | | | | | | | | | | | | | | | | | | | | | | | | | | | | | | | |  | |  |
| ** | | labs whose results are reported in mg/ml | | | | | | | | | | | | | | | | | |  | | | | |  | | | |  | | |  | | | |  | | | |  | |  |
| *** | | excluding lab 17 as only 1 assay performed | | | | | | | | | | | | | | | | | |  | | | | |  | | | |  | | |  | | | |  | | | |  | |  |

^1^Reported titers in reciprocal dilutions for sera and in ng/ml for the A-Q samples; Ranking is based on the GM obtained for the ELISA vs IH (high to low

**Table 3. Information on the determination of the cut-offs of the assays, where available**

| **Lab** | **Approach for cut-off determination** |
| --- | --- |
| **1a** | 8 AU/ml; mean anti-infliximab ADA + 20SD, established using 40 infliximab-naïve patients with different pathologies |
| **2a** | 10 ng/ml; - kit (estimated using 152 healthy patient samples, 95th percentile) |
| **3** | 10 ng/ml - kit (estimated using 152 healthy patient samples, 95th percentile) |
| **4** | LLOQ - 2.5 ng/ml |
| **5** | kit cut-off: mean of '0' standard + 2SD |
| **6** | 10 AU/ml = OD cut-off control; established by diluting a highly positive sample until no more linear dilution possible. 40 infliximab-naïve samples tested with this cut-off: 97.5% neg |
| **7** | 10 AU/ml = OD cut-off control; established by diluting a highly positive sample until no more linear dilutions possible. 40 infl-naïve samples tested with this cut-off: 97.5% neg |
| **8a** | no formal validation – in-house testing for research purposes only; Mean of blank + 2SD |
| **9** | 50 U/ml (equivalent to 50ng/ml) |
| **10** | floating cut point: NC of plate x normalization factor |
| **11** | floating cut point (average 8 NC in each plate x N factor); normalization factor determined to have 5% false positive rate in normal human serum |
| **12** | relative ECL -1.32 for RA matrix and 1.35 for IBD; determined using min 30 negative sera; non-parametric method (95th percentile) |
| **13** | 41 individual serum samples analysed against the NC pool. 5% non-parametric CP determined. Study CP = 1.27. Plate specific CP in RLU (mean NC x study CP) |
| **17a** | ADA positive sample: response ≥ Mean of blank * cut-point; cut-point is determined statistically per drug-naive population assuming a predefined false positive rate |
| **15** | LoD 0.2μg/ml |
| **1b** | LoD 23AU/ml |
| **2b** | 10 ng/ml; population of serum samples from healthy donors or untreated patients; 95th percentile |
| **14** | Mean + 6 SD of 100 pretreatment values |
| **8b** | no formal validation – in-house testing for research purposes only |
| **17b** | NAb positive sample: response ≤ Mean of Control * 0.87 (cut-point); cut-point is determined statistically per drug-naive population assuming a predefined false positive rate |
| **8c** | no formal validation - – in-house testing for research purposes only |
| **16** | Threshold= threshold factor x normalised TNF activity of reference. Serum samples from 50 treatment-naïve patients (25 RA & 25 Crohn's) were tested; threshold factor calculated at 99.5%CI. |

Table 4: Summary of results from accelerated temperature degradation studies of lyophilized preparations A and B

| Method | Sample | Time stored (years) | Storage Temperature (°C) | LCL | Relative Potency to -20°C | UCL |
| --- | --- | --- | --- | --- | --- | --- |
| ELISA - binding | 19/232 | 1.917 | -70 | 95.8% | 98.4% | 101.0% |
| ELISA - binding | 19/232 | 1.917 | +4 | 95.0% | 97.5% | 100.1% |
| ELISA – binding | 19/232 | 1.917 | +20 | 94.2% | 97.6% | 101.1% |
| ELISA – binding | 19/232 | 1.917 | +37 | 85.2% | 90.7% | 96.5% |
| ELISA - binding | 19/232 | 1.917 | +45 | 81.0% | 98.1% | 118.7% |
| ELISA – binding | 19/234 | 1.833 | -70 | 94.8% | 99.4% | 104.2% |
| ELISA – binding | 19/234 | 1.833 | +4 | 94.0% | 98.1% | 102.3% |
| ELISA – binding | 19/234 | 1.833 | +20 | 95.1% | 98.6% | 102.2% |
| ELISA - binding | 19/234 | 1.833 | +37 | 90.7% | 95.7% | 101.0% |
| HEKbl – neutr | 19/232 | 1.917 | -70 | 95.4% | 97.6% | 99.8% |
| HEKbl - neutr | 19/232 | 1.917 | +4 | 95.3% | 99.3% | 103.5% |
| HEKbl - neutr | 19/232 | 1.917 | +20 | 93.1% | 96.9% | 100.8% |
| HEKbl - neutr | 19/232 | 1.917 | +37 | 91.0% | 93.2% | 95.5% |
| HEKbl - neutr | 19/232 | 1.917 | +45 | 59.5% | 66.1% | 73.3% |
| HEKbl - neutr | 19/234 | 1.833 | -70 | 99.0% | 101.6% | 104.3% |
| HEKbl - neutr | 19/234 | 1.833 | +4 | 98.4% | 102.1% | 106.1% |
| HEKbl - neutr | 19/234 | 1.833 | +20 | 96.7% | 98.8% | 101.0% |
| HEKbl - neutr | 19/234 | 1.833 | +37 | 93.5% | 95.6% | 97.8% |
| HEKbl - neutr | 19/234 | 1.833 | +45 | 80.2% | 85.3% | 90.6% |

ELISA 19/232 geometric mean potency derived from 6 estimates for +45 and 9 estimates in all other cases

ELISA 19/234 geometric mean potency derived from 6 estimates in all cases

HEKbl 19/232 geometric mean potency derived from 12 estimates for -20 and 9 estimates in all other cases

HEKbl 19/234 geometric mean potency derived from 12 estimates for -20, 6 estimates for +45 and 9 estimates in all other cases; LCL and UCL: Lower and Upper 95% confidence limits

Table 5: Summary of results from freeze-thaw studies of lyophilized preparations lyophilized preparations relative to a fresh ampoule

| Method | Sample | Number of freeze/thaw cycles | LCL | Relative Potency, % | UCL |
| --- | --- | --- | --- | --- | --- |
| ELISA - binding | 19/232 | 1x | 93.7% | 97.5% | 101.4% |
| ELISA - binding | 19/232 | 2x | 93.5% | 98.2% | 103.1% |
| ELISA - binding | 19/232 | 3x | 93.1% | 97.0% | 101.0% |
| ELISA - binding | 19/232 | 4x | 91.6% | 96.6% | 101.9% |
| ELISA - binding | 19/234 | 1x | 79.5% | 102.7% | 132.7% |
| ELISA - binding | 19/234 | 2x | 79.2% | 99.1% | 124.0% |
| ELISA - binding | 19/234 | 3x | 83.8% | 101.4% | 122.7% |
| ELISA - binding | 19/234 | 4x | 88.3% | 98.1% | 109.0% |
| HEKbl - neutr | 19/232 | 1x | 99.2% | 103.7% | 108.3% |
| HEKbl - neutr | 19/232 | 2x | 85.4% | 103.7% | 126.0% |
| HEKbl - neutr | 19/232 | 3x | 93.3% | 102.7% | 112.9% |
| HEKbl - neutr | 19/232 | 4x | 85.5% | 99.2% | 115.2% |
| HEKbl - neutr | 19/234 | 1x | 95.1% | 98.1% | 101.1% |
| HEKbl - neutr | 19/234 | 2x | 89.0% | 98.5% | 109.0% |
| HEKbl - neutr | 19/234 | 3x | 92.2% | 99.3% | 106.9% |
| HEKbl - neutr | 19/234 | 4x | 94.9% | 99.7% | 104.7% |

Geometric Mean potency derived from 7 estimates for ELISA 19/232 and 4 estimates in all other cases;

LCL and UCL: Lower and Upper 95% confidence limits
